# Structure-tuned membrane active Ir-complexed oligoarginine overcomes cancer cell drug resistance and triggers immune responses in mice†

**DOI:** 10.1039/d0sc03975f

**Published:** 2020-08-10

**Authors:** Shuangshuang Ji, Xiuzhu Yang, Xiaolong Chen, Ang Li, Doudou Yan, Haiyan Xu, Hao Fei

**Affiliations:** School of Nano-Tech and Nano-Bionics, University of Science and Technology of China Hefei 230026 PR China hfei2008@sinano.ac.cn; CAS Key Laboratory of Nano-Bio Interface, Division of Nanobiomedicine, Suzhou Institute of Nano-Tech and Nano-Bionics, Chinese Academy of Sciences Suzhou 215123 PR China; Institute of Basic Medical Sciences, Chinese Academy of Medical Sciences & Peking Union Medical College Beijing 100005 PR China

## Abstract

The development of chemotherapy, an important cancer treatment modality, is hindered by the frequently found drug-resistance phenomenon. Meanwhile, researchers have been enthused lately by the synergistic use of chemotherapy with emerging immunotherapeutic treatments. In an effort to address both of the two unmet needs, reported herein is a study on a series of membrane active iridium(iii) complexed oligoarginine peptides with a new cell death mechanism capable of overcoming drug resistance as well as stimulating immunological responses. A systematic structure–activity relationship study elucidated the interdependent effects of three structural factors, *i.e.*, hydrophobicity, topology and cationicity, on the regulation of the cytotoxicity of the Ir(iii)-oligoarginine peptides. With the most prominent toxicities, Ir-complexed octaarginines (R8) were found to display a progressive oncotic cell death featuring cell membrane-penetration and eruptive cytoplasmic content release. Consequently, this membrane-centric death mechanism showed promising potential in overcoming multiple chemical drug-resistance of cancer cells. More interestingly, the eruptive mode of cell death proved to be immunogenic by stimulating the dendritic cell maturation and inflammatory factor accumulation in mice tumours. Taking these mechanisms together, this work demonstrates that membrane active compounds may become the next generation chemotherapeutics because of their combined advantages.

## Abstract 2

Structure optimized Ir-complexed cyclic octa-arginine shows a potential of “one-drug two-effects” for cancer treatment.

## Graphical abstract

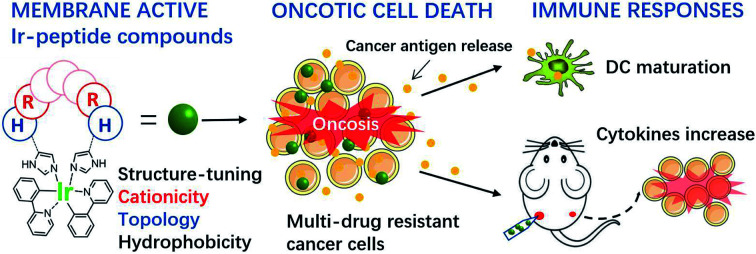

## Introduction

In recent years, as the understanding of cancer and its interaction with the immune system has deepened, cancer immunotherapy that attacks tumour cells by stimulating the body's own immune system has developed rapidly, which has had a profound impact on the paradigms of cancer treatment.^1,2^ However, immunotherapy also has its limitations, such as high treatment costs, large individual variances, and common immune-toxic effects such as cytokine storms, *etc.*^3,4^ Inexpensive chemotherapy remains a key part in clinical cancer therapy, and recently, some studies have shown that chemotherapy drugs can also activate the patient's own anti-tumour immune response by stimulating the body's inflammatory response, and producing a synergistic effect with immunotherapy methods.^5–7^ However, chemotherapy is facing severe drug resistance in clinical practice. As the immune-stimulating effects of chemotherapy drugs depend on tumour cell sensitivity to the agent,^8^ the subsequent immune effects may become silent in drug-resistant cancer cells. Moreover, contrary to the highly dynamic and heterogeneous mutational characteristics of cancer,^9,10^ the mechanisms of traditional chemotherapy drugs are mostly limited to targeting DNA or the proteins directly involved in cell proliferation, and it has become difficult to meet the increasing need for diversified treatment options. These excessively restricted mechanisms of action also promote clinical chemotherapy resistance to certain extent.^11^ Thus, it is necessary and urgent to develop chemotherapeutic agents with novel mechanisms of action to overcome such limitations and for usage beyond the traditional scope.

The plasma membrane is an important target for many cell killing mechanisms that exist in nature. For example, recently elucidated inflammatory cell death programs such as necroptosis and pyroptosis are executed by plasma membrane pore forming proteins of phosphorylated MLKL or truncated gasdermins, respectively.^12–15^ Studies have further exploited the potential of enhancing anti-tumour immunity *via* plasma membrane targeted killing strategies.^15–17^ Because the plasma membrane is functionally indispensable, uniform in structure and unrelated to genetic mutations, it is also believed to be a target for overcoming drug resistance in cancer. Similarly using membrane-targeted mechanisms, a class of membrane active peptides can cause cell membrane lysis or penetration by interacting with membrane phospholipids and glycans *via* hydrophobic and electrostatic forces.^18–22^ However, membrane active peptides face development challenges shared by all the peptide-based pharmaceuticals. Artificial modifications such as lipidation, cyclization and stereoisomerisation are often required to overcome the shortcomings inherent to peptides, including their natural hydrophilicity, structural instability and proteolytic vulnerability.^23–29^

Meanwhile, a notable class of membrane-targeting cyclometalated iridium(iii) complexes, have gained increasing attention because of their biomolecular reactivities and potent anticancer activities of novel mechanisms.^30–37^ In this work, these two classes of membrane active compounds are bioconjugated to remove their respective disadvantages; for example, the iridium complex contributes to the peptide structural stability and enables luminescence-based analysis, whereas peptides endow the iridium complex with a suitable solubility. This combination may also provide structural insights on new compounds' membrane activity and therapeutic mechanisms beyond our previous understanding.^38,39^

**Fig. 1:**
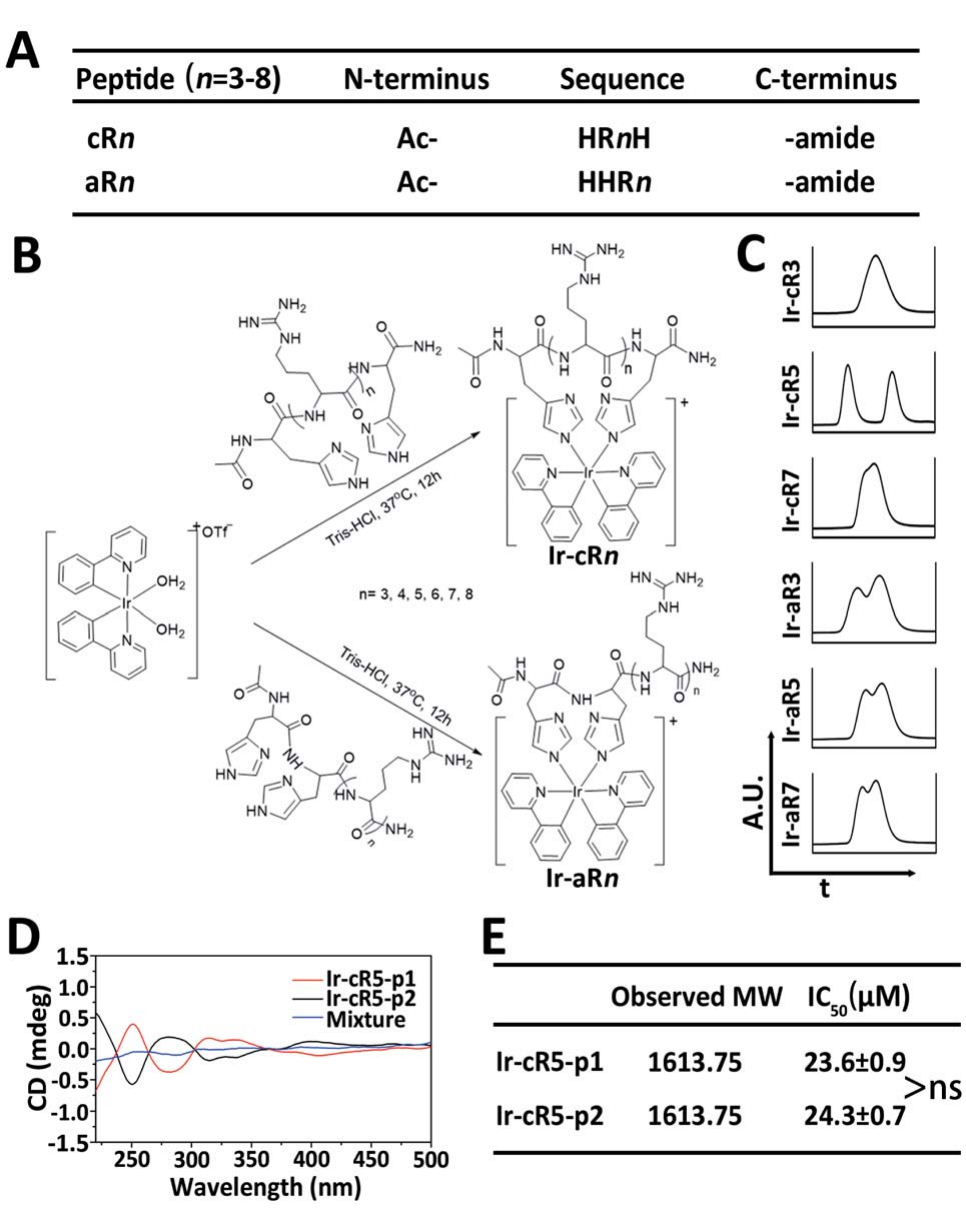
(A) Abbreviated list of peptide sequences. (B) Synthesis routes of Ir-peptides. (C) Intercepted UPLC spectra of selected Ir-peptides. (D) CD spectra of the two components of Ir-cR5 and their 1 : 1 (*n*/*n*) mixture. (E) Observed molecular weights and IC_50_ of two components of Ir-cR5, ns: not significant.

Oligoarginines, as cell penetrating peptides, have been well-studied in the past several decades because of their capacity to carry ‘cargos’ into cells.^40,41^ It has also been noticed that when an arginine-rich peptide is combined with hydrophobic moieties it can lead to significant cytotoxicity which has rarely been reported in systematic studies.^42^ In this study, peptides of oligoarginines were chosen to conjugate with Ir(ppy)_2_ (bis(2-phenylpyridine)iridium(iii)) complexes (abbreviated as Ir-peptides). The relationship between the physicochemical properties and the corresponding cell activities have been questioned. Cationicity, hydrophobicity and topology were three factors used to regulate the activity of the Ir-peptides. As illustrated in Fig. 1A, peptide cationicity was varied from 3 to 8 by modulating the number of arginines. Two histidines were appended as Ir-coordination positions. Peptides were denoted as aR*n* if the two histidine residues were appended on the amino-termini of the peptides, or cR*n* if histidines were appended on both the amino- and the carboxyl-termini for peptide cyclization (*n* indicates the number of arginine residues). Linear or cyclic topology of Ir-peptides can be obtained when the hydrophobic Ir(ppy)_2_ group was introduced to aR*n* or cR*n* through the iridium–histidine interaction, the coordinated aR*n* or cR*n* was denoted as Ir-aR*n* or Ir-cR*n*, and their synthesis routes are summarized in Fig. 1B.

## Results and discussion

The success of coordination of aR*n* or cR*n* with the Ir(ppy)_2_ group was first indicated by the generation of a phosphorescence emission by the Ir-peptides. All the Ir-aR*n* and Ir-cR*n* peptides showed the characteristic emission profile of Ir(iii)–histidine(s) luminophore(s) under 328 nm excitation (Fig. S2, ESI†). The molecular weight and purity of the Ir-peptides were further confirmed by electrospray ionization mass spectra (ESI-MS) and ultrahigh performance liquid chromatography (UPLC) analysis (Fig. S3–S31, ESI†). Interestingly, the UPLC spectra of Ir-peptides exhibited three types of elution profiles: two peaks with equal areas, two overlapping peaks or a single peak (Fig. 1C). In an established synthesis route *via* a μ-chloride dimer, it is common to find the Ir-complex products as racemic mixtures with the ratio of Δ and Λ isomers being 1 : 1.^43,44^ To verify that the Ir-peptides were racemic mixtures, the two peak components of Ir-cR5 were further separated and named Ir-cR5-p1 and Ir-cR5-p2, respectively. The ESI-MS revealed identical molecular weights for Ir-cR5-p1 and Ir-cR5-p2 (Fig. 1E and S15, ESI†). Circular dichroism (CD) measurements (220–500 nm) indicated that Ir-(ppy)_2_ adducts of Ir-cR5-p1 and Ir-cR5-p2 were indeed mirror-image enantiomers, and the 1 : 1 mixture of Ir-cR5-p1 and Ir-cR5-p2 showed neutral chirality (Fig. 1D). The CD spectra of Ir-cR3, Ir-cR7, Ir-aR3, Ir-aR5 and Ir-aR7 all showed similar profiles as the Ir-cR5 mixture, indicating the racemic nature of the two series of Ir-peptides (Fig. S32, ESI†). More importantly, use of a MTT assay revealed that Ir-cR5-p1 and Ir-cR5-p2 have the same cytotoxic activities against HeLa cells (Fig. 1E), confirming that the chiral status of the Ir(ppy)_2_ group does not play a role in the membrane activity of Ir-peptides.

**Fig. 2:**
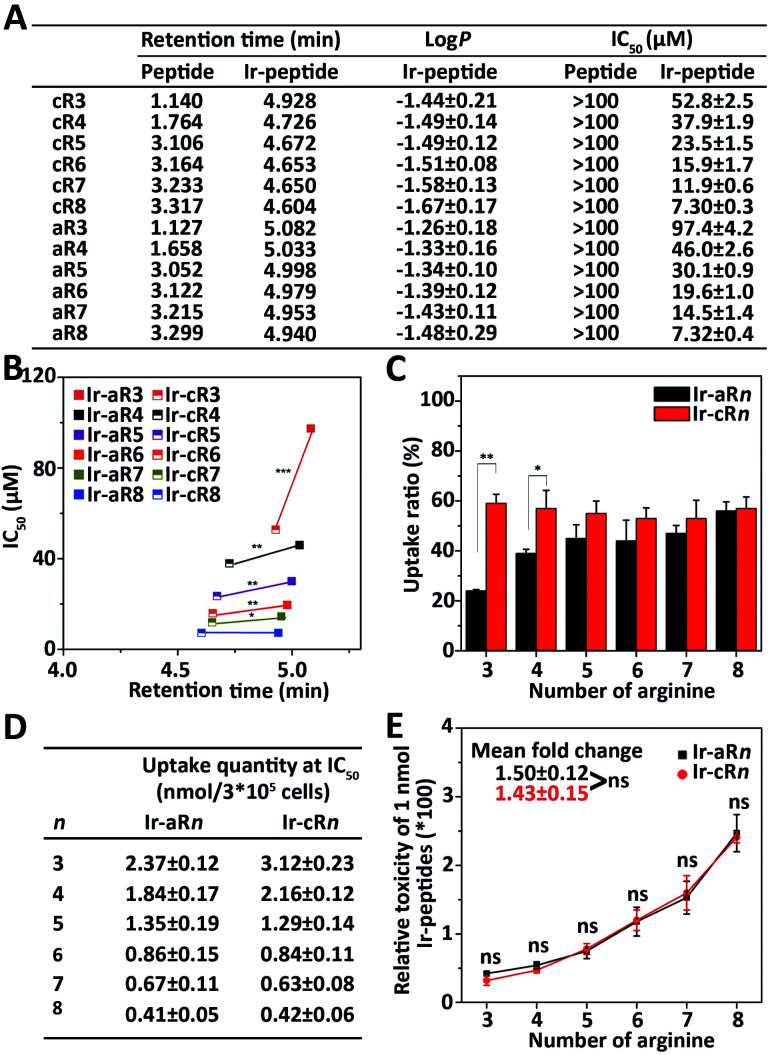
(A) Retention times and IC_50_ values of oligoarginine peptides and Ir-peptides, log *P* values of Ir-peptides. (B) Cytotoxicity and hydrophobicity difference between Ir-aR*n* and Ir-cR*n*. *P < 0.05, **P < 0.01, ***P < 0.001. (C) Comparison of uptake ratios of Ir-aR*n* and Ir-cR*n* at concentrations of 0.5 × IC_50_ in HeLa cells. *P < 0.05, **P < 0.01. (D) Calculated uptake quantity at the concentration of IC_50_ of Ir-peptides. (E) Calculated relative toxicity of 1 nmol Ir-peptides. Mean fold change was obtained by averaging the toxicity ratios between Ir-peptide *n* + 1 and *n*. ns: not significant.

Because the Ir(ppy)_2_ group is hydrophobic and the oligoarginine peptides are hydrophilic, the newly constructed Ir-peptides should show varied gross hydrophobicity. For comparison purposes, the hydrophobicity was assessed by UPLC using retention time (RT) as a reference. The log *P* values indicating the water/octanol partition of the compounds were also determined. As shown in Fig. 2A, the RTs of the unlabelled oligoarginine peptides ranged from 1.127 to 3.317 min, whereas after Ir coordination, the RTs of all Ir-peptides greatly increased to a range above 4.6 min. The determined log *P* values (Fig. 2A) also suggested a similar trend of hydrophobicity change within the set of compounds. Interestingly, linear Ir-peptides generally have a higher hydrophobicity than the cyclic forms, possibly because of a more exposed Ir(ppy)_2_ group in the linear form. These together indicate that the Ir(ppy)_2_ group contributes to the majority of the increase of the Ir-peptides' hydrophobicity. Next, an investigation was carried out on how the change of hydrophobicity affected the cytotoxicity of the Ir-peptides. In contrast to the [Ir(ppy)_2_(H_2_O)_2_]OTf or oligoarginine peptides' unmeasurable toxicity (IC_50_ > 100 μM, Fig. 2A and S34, ESI†), all of the Ir-peptides' cytotoxicity was distinctly enhanced upon Ir coordination (Fig. 2A). Photo-toxicity has been sometimes observed in other cyclometalated iridium(iii) complexes.^45,46^ To exclude any involvement of photo-toxicity from the Ir-complex in the MTT assay, the ability of the Ir-peptides to generate reactive oxygen species (ROS) was examined by a ROS probe, 9,10-anthracenediyl-bis(methylene)-dimalonic acid (ABDA), under the lighting of a biosafety hood (44 mW cm^−2^) for 30 min to mimic the sample light exposure during the MTT assay and no effects of photo-toxicity could be detected (Fig. S35, ESI†). This suggested that the increased gross hydrophobicity was generally responsible for the increase of the Ir-peptides' cytotoxicity. A close comparison of the relationship of RT and cytotoxicity was made by plotting the two factors as shown in Fig. 2B. It revealed that within the series of Ir-aR*n* or Ir-cR*n*, as the number of arginine residues increased, the RT exhibited a slight decrease whereas cytotoxicity gradually increased. Interestingly, across the series, it appeared that the more hydrophobic Ir-aR*n* were almost always less toxic than Ir-cR*n*, especially for the peptides that have fewer numbers of arginines. This implies that factors other than hydrophobicity were influencing the cytotoxicity of the Ir-peptides.

The significant differences between the Ir-peptides were their topology and cationicity. Studies have reported that cyclization of peptides could enhance their endocytosis and also that the number of guanidine groups was related to the peptides' cell uptake efficiency.^47–49^ The cationicity and topology difference may influence the Ir-peptides' cellular uptake thus causing the notable toxicity difference. To verify this, the luminescent nature of Ir-peptides was used to quantitatively determine the cell uptake ratios of Ir-peptides based on the total input and the residual amounts, both of which were calculated against calibrated standard curves (Fig. S36, ESI†). The results showed that whereas the uptake ratios of Ir-cR*n* were all consistently above 50%, the Ir-aR*n* exhibited a varied ratio from 24% to 56% as the arginine numbers grew from 3 to 8 (Fig. 2C). The gap between the uptake ratios of Ir-aR*n* and Ir-cR*n* gradually diminished as the arginine number increased. These data indicated that cyclic conformations have advantages over linear conformations for cell entry of the Ir-peptides, and this may be due to the reduced flexibility leading to more consistent and effective interaction of Ir-cR*n* with cell membrane than Ir-aR*n*. However, when the arginine number reached 8, both Ir-aR8 and Ir-cR8 showed similar cell uptake, suggesting the flexibility of linear structure may have been overwhelmed by a stronger electrostatic interaction between octaarginine and the cell membrane.

These results suggested that the topology effect on the different uptake efficiencies may have partially compensated for the previously observed opposite tendency between the hydrophobicity and toxicity across the linear and the cyclic series. To confirm this, the quantity of Ir-peptides taken up by cells at each Ir-peptide's IC_50_ concentration was calculated using the uptake ratios obtained previously (Fig. 2D). The “relative molecular toxicity” was further derived by dividing the killing efficiency (set as 100) by the amount of each Ir-peptide (in nmole) that cells uptake at the IC_50_ concentration. As shown in the data plots in Fig. 2E, Ir-aR*n* and Ir-cR*n* exhibited an undistinguishable dependency of molecular toxicity on the number of arginines.

Together, these analyses demonstrated that adding the hydrophobic Ir(ppy)_2_ enhanced the oligoarginine's cytotoxicity in general, but finer regulation could be achieved by adjusting the complexes' topology and cationicity. Cyclic structures were more advantageous because of their consistency in cell uptake especially when the arginine number was small and the molecular toxicities of the Ir-peptides were quantitatively dependent on the number of arginines. Thus, every physicochemical property including hydrophobicity, cationicity and topology played a conditional role in interdependently determining the cytotoxicity of the Ir-peptides.

**Fig. 3:**
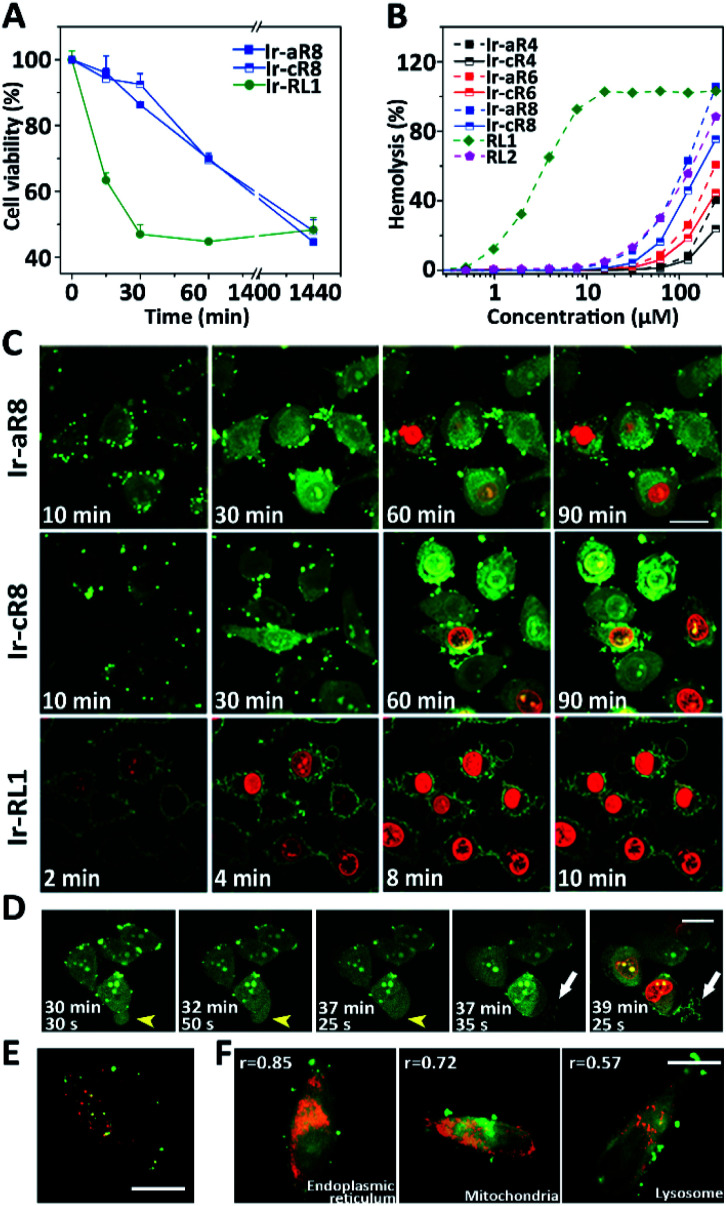
(A) Viability of HeLa cells treated with Ir-cR8, Ir-aR8 or Ir-RL1 at IC_50_ concentrations for various amounts of time. (B) Haemolytic percentage of erythrocytes treated with various peptides. (C) CLSM observations on morphological evolution and membrane leakage of HeLa cells treated with Ir-aR8, Ir-cR8 or Ir-RL1 (green) at IC_50_ concentrations and PI (red), scale bar: 20 μm. (D) Time-series CLSM of the cell death process caused by 10 μM Ir-cR8 (green) co-stained with PI (red). HeLa cells underwent changes from bubbling to swelling (yellow arrowheads), and membrane rupture with eruptive content leakage (white arrows), scale bar: 20 μm. (E) CLSM image of HeLa cells incubated with 5 μM Ir-cR8 (green) and 250 μg mL^−1^ Dextran-RhB (red), scale bar: 15 μm. (F) CLSM images of HeLa cells pre-stained with LysoTracker Red DND-99, MitoTracker Red, ER-Tracker Red followed by incubation with 5 μM of Ir-cR8 (green), *r*: Pearson's correlation coefficient, scale bar: 20 μm.

Given their potent toxicity among the Ir-peptide series, Ir-aR8 and Ir-cR8 were examined further to determine their cell killing mechanisms in detail. To better clarify the cell killing kinetics, time-lapse MTT assays were carried out to assess cell viabilities of HeLa cells treated with Ir-aR8 or Ir-cR8 at their respective IC_50_ concentrations at different times (Fig. 3A). A previously reported, Ir-labelled control peptide RL1, which is a typical membrane-lytic α-helical amphipathic peptide,^42^ was also included and displayed a rapid killing effect resulting in over 50% cell death within 30 min. In contrast, both Ir-aR8 and Ir-cR8 showed much slower killing kinetics, causing about 30% cell death in 1 h. The haemolysis assay showed that these peptides had a weaker haemolytic capacity similar to that of the apoptotic peptide RL2 (ref. 42) compared to the membrane-lytic peptide RL1 (Fig. 3B). The slower killing kinetics and the weaker haemolytic ability of these peptides suggest that is an alternative mechanism of action from the membranolytic one.

The inherent luminescence from the Ir-labelling allowed the use of confocal laser scanning microscopy (CLSM) to observe the entire process of cell entry, cell morphological changes, and ultimate cell death in Ir-peptide treatment. As a control (Fig. 3C), Ir-RL1 exhibited rapid uniform accumulation on the cell membrane, causing membrane bubbling and disruption, which was indicated by complete propidium iodide (PI) staining of the cell nuclei within 10 min. In contrast, both Ir-aR8 and Ir-cR8 were initially found to accumulate at discrete spots on the cell surface and later penetrated into the cytoplasm. The treated cells first experienced apparent cell rounding and size expansion, and then slowly progressed to plasma membrane disruption over a period of more than 1 h. A more detailed time-series of microscopic images demonstrated the dynamic death process of HeLa cells incubated with Ir-cR8 as shown in Fig. 3D (and for Ir-aR8 see Fig. S37, ESI†). After an initial Ir-cR8 incubation period, the cell membrane began to form bubbles, which in the next a few minutes fused back into the cell membrane causing a swollen appearance of the cell (Fig. 3D, highlighted by yellow arrowheads and also in other cells). This was then followed by subsequent cell membrane rupture and massive eruptive leakage of the cellular content (Fig. 3D, highlighted by white arrows and also in other cells), together with the penetration of PI through the broken site of the cell membrane and progressive staining of the nucleus from partial to full. The sequential onset of morphological events of cell membrane bubbling, swelling, and its eruptive cell content leakage appeared to be consistent with the manifestation of oncosis.^50^

In order to determine the potential cellular target related to this death mode, the uptake pathways and subcellular distribution of Ir-cR8 were explored. Macropinocytosis has been reported to be one of the important pathways in the endocytosis of arginine-rich peptides;^51^ however, the results of this work suggested that Ir-cR8 could also enter cells through an energy-independent pathway and not much overlap of Ir-cR8 with dextran-RhB (a fluorescently-labelled micropinosome marker) was observed (Fig. 3E, S38A and S38B, ESI†). In addition, the spot morphology that Ir-cR8 exhibited on the cell membrane resembled the nucleation zones or particle structures reported previously.^52,53^ These data together suggested a direct interaction on the cell membrane and passive penetration *via* fusion of Ir-cR8 when entering the cell.^54^ Next, the intracellular distribution of Ir-cR8 was explored by CLSM (Fig. 3E and S38C, ESI†). The Pearson's correlation coefficient of Ir-cR8 with different markers showed that, although diffused in the cell, Ir-cR8 preferred to distribute in the endoplasmic reticulum and the mitochondria rather than in the lysosomes, a localisation potentially having correlation with the oncosis-like cell death.^55^

**Fig. 4:**
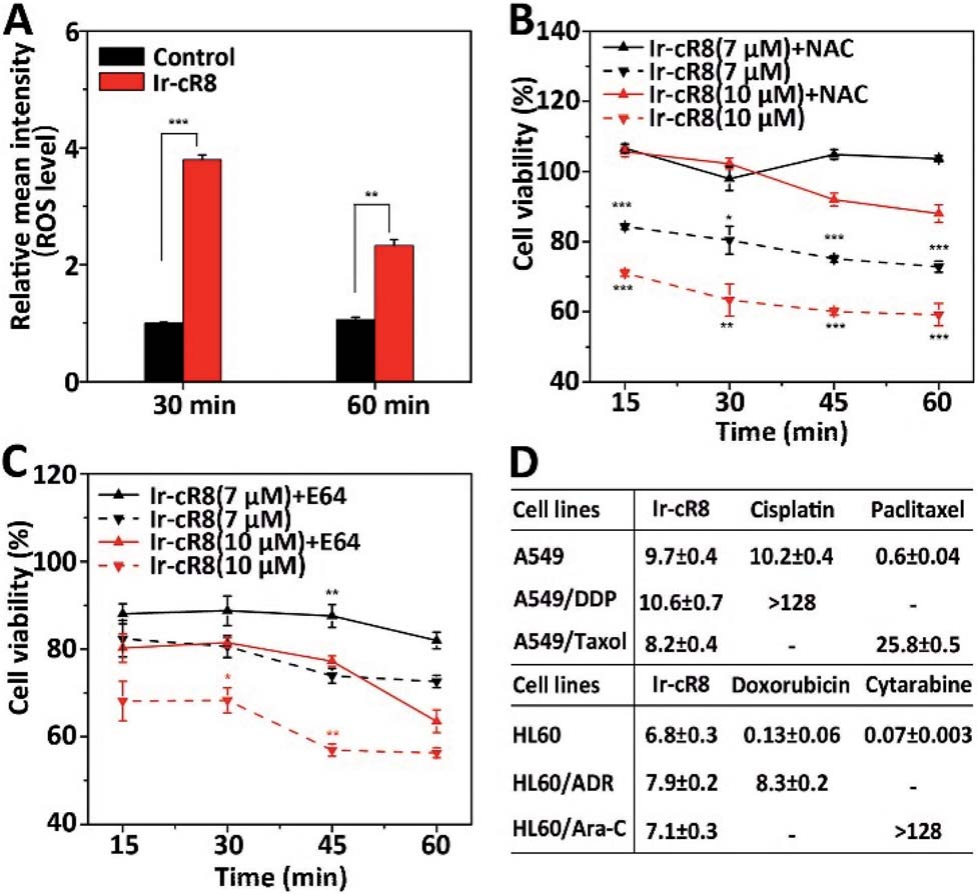
(A) Change of ROS level in HeLa cells treated with Ir-cR8 at its IC_50_ concentration for the indicated times, **P < 0.01, ***P < 0.001. (B) Viability of HeLa cells treated with Ir-cR8 (7, 10 μM) in an hour with/without ROS inhibitor NAC (10 mM), *P < 0.05, **P < 0.01, ***P < 0.001. (C) Cytotoxicity change induced by Ir-cR8 (7, 10 μM) in an hour with/without calpain inhibitor E64 (15 μM), *P < 0.05, **P < 0.01. (D) Antiproliferative activity of Ir-cR8, cisplatin (DDP), paclitaxel (Taxol), doxorubicin (ADR) and cytarabine (Ara-C) towards the parent or the corresponding drug-resistant cancer cells.

Next, the intracellular molecular mechanisms that may be associated with this mode of death were investigated. As the signalling pathways that regulate oncosis have not been fully elucidated, circumstantial evidence was searched to verify whether the cell death caused by the Ir-peptides was of oncotic nature. The excessive generation of intracellular ROS has been associated with several forms of cell death including apoptosis,^56^ ferroptosis,^57^ necroptosis^58^ and oncosis.^59^ Therefore, the change of intracellular ROS was monitored first. Results showed that Ir-cR8 could induce cell ROS generation by nearly 4-fold compared to the control after 30 min and more than 2-fold at 1 h (Fig. 4A). The induced ROS production was consistent with the subcellular enrichment of Ir-cR8 in two ROS-related organelles the endoplasmic reticulum and mitochondria as described previously.^60,61^ Further analysis indicated that a ROS scavenger agent *N*-acetyl-l-cysteine (NAC) could significantly reverse the cytotoxicity induced by Ir-cR8 in short (15–60 min, Fig. 4B) or long (24 h, Fig. S39, ESI†) assays. Next, a series of specific inhibitor assays were applied to distinguish the involvement of different pathways. The caspases are well-known as key players in apoptosis, and also in mediating pyroptosis.^62–64^ However, the results of the caspase-3 activity assay and the Z-VAD-FMK pan-caspase inhibition assay showed no correlation to Ir-cR8 induced cell death (Fig. S40–S42, ESI†). Whether Ir-cR8 cytotoxicity involved the pathways of pyroptosis,^15^ ferroptosis^65^ or necroptosis^66^ was further examined using specific inhibitors: 3,4-dichloroisocoumarin, ferrostatin-1 or necrostatin-1, respectively. Results suggested that Ir-cR8 did not cause cell death *via* these pathways (Fig. S43–S46, S48 and S49†). Finally, because several studies have reported the promotive function of calpains in oncosis,^67^ a pan calpain inhibitor E64 was used to assess the role of calpain in Ir-cR8 induced cytotoxicity. Interestingly, the results indicated that the inhibition of calpain apparently improved cell viability within 1 h of Ir-cR8 treatment (Fig. 4C), confirming the involvement of calpain, although the results of a long treatment time test (24 h, Fig. S47, ESI†) with E64 suggested that sole inhibition of calpain was insufficient to reverse an Ir-cR8 induced death course of HeLa cells. Thus, after excluding several major pathways of cell death currently known, the confirmed calpain involvement and the observed morphological characteristics corroboratively point to a death mechanism of oncosis of Ir-cR8 treated HeLa cells.

The mode of action *via* which these membrane active Ir-peptides interact with the cell membrane has no overlap with the chemical therapeutics currently approved for clinical use, and some studies have also reported that oncosis may have a significant advantage in overcoming the drug-resistance in cancer cells.^32^ To assess the potential of Ir-peptides in overcoming drug-resistance, the cytotoxicity of Ir-cR8 in cancer cell lines was compared especially in those resistant to four mainstream chemotherapy drugs: cisplatin, paclitaxel, doxorubicin and cytarabine. Whereas all four drugs showed drastically weakened toxicity in the drug-resistant cells compared to their corresponding parent cells, the IC_50_s of Ir-cR8 were all close for both the parent and the drug-resistant cancer cell lines (Fig. 4D), showing its consistent killing capacity with the promise of overcoming traditional chemotherapy drug resistance. In addition, the cytotoxicity of Ir-cR8 to human embryonic kidney (HEK) 293T cells and human umbilical vein endothelial cells (HUVEC) was also tested. The IC_50_s of these two normal cell lines were 17.1 and 24.7 μM, respectively (Fig. S50, ESI†). The cytotoxicity difference of Ir-cR8 towards normal cell lines and cancer cell lines may result from the more negative charges in the membrane surface of the cancer cells than in normal cells, which induced a stronger electrostatic interaction between Ir-cR8 and the cancer cell membrane.^68^

**Fig. 5:**
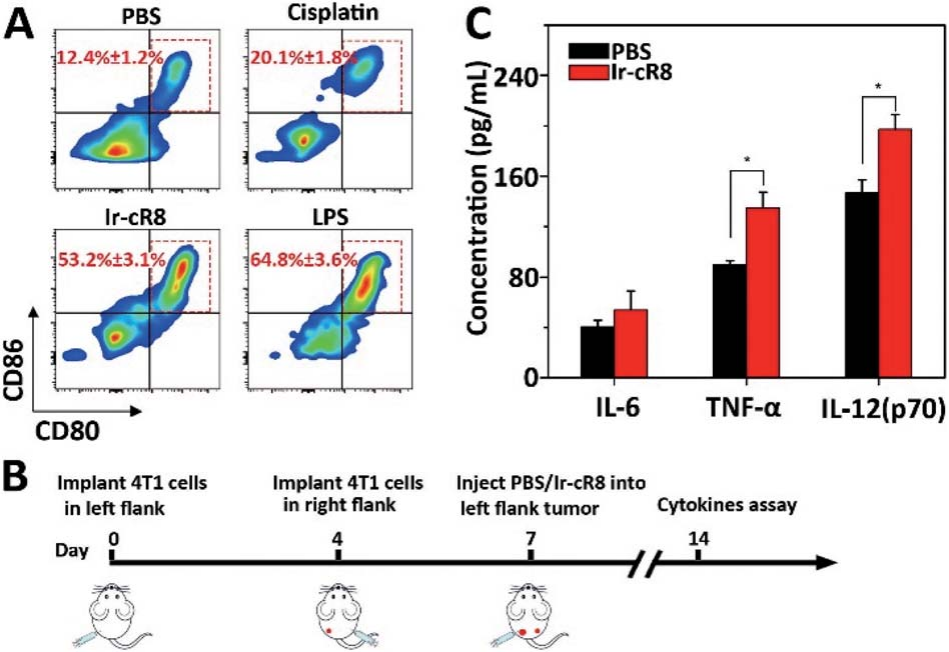
(A) DC maturation (CD11c^+^CD80^+^CD86^+^) when treated with LPS or supernatant of 4T1 cells incubated with PBS, cisplatin, or Ir-cR8. (B) Treatment schedule: 4T1 cells were implanted into left and right flank on day 0 and day 4, respectively, and PBS or Ir-cR8 (0.1 μmol) was injected into the tumour of the left flank on day 7. The tumours of the right flank were collected on day 14 for cytokine assay. (C) Cytokine level in right flank tumour from mice (*n* = 3) at day 14, *P < 0.05.

More interestingly, the Ir-cR8 treated cancer cells manifested oncotic cell death which was likely to be a form of immunogenic cell death because of its eruptive leakage of cell content.^69^ Dendritic cells (DCs) are crucial antigen-presenting cells and play key roles in initiating and regulating innate and acquired immunities, thus they have great potential for inducing efficient anti-tumour immunity by manipulating the DCs.^70^ It is supposed that by exposure to the cancer antigens ejected by oncosis, immature DCs would engulf and process the antigen and then transform into mature DCs. To verify this, mouse bone marrow derived dendritic cells (BMDCs) were incubated with the supernatant of 4T1 cells treated with PBS, cisplatin or Ir-cR8, and with lipopolysaccharide (LPS, a potent innate immune response stimulator) as a positive control. Results of flow cytometry analysis suggested that, compared with cisplatin, a known less immunogenic cell death inducing agent,^5^ Ir-cR8 induced cell death distinctly promoted *in vitro* maturation of DCs, to a level close to that of LPS, as indicated by the presence of CD80 and CD86 on the mature DC cell surface (Fig. 5A). Cytokines also played an important part in regulating immune response. Three cytokines were chosen for investigation, IL-6, TNF-α and IL-12(p70), because IL-6 is an important cytokine for B cell stimulation in humoral immunity, TNF-α is a typical marker for cellular immunity and can kill cancer cells directly, and IL-12(p70) can stimulate the proliferation of activated T cells and induce the cytotoxic activity of the CTL and NK cells.^71–74^ A bilateral tumour model was built to assay the anti-tumour immune activation following the schedule shown in Fig. 5B. The left flank tumour had PBS or Ir-cR8 injected into it as a reservoir of tumour antigens, and the cytokine concentrations in the right flank tumour were used to evaluate the effect of the immune stimulation. The upregulation of cytokines in right-flank tumour of mice treated with Ir-cR8 (Fig. 5B) revealed the activation of innate immunity and tumour-specialized cellular immunity.

## Conclusions

In order to systematically explore the medicinal potential of membrane active agents for the treatment of cancers, a model system comprising a series of Ir-complexed oligoarginine compounds was established and then hydrophobicity, topology and cationicity, being the three main factors that affect cytotoxicity, were studied. Importantly, the less haemolytic and membrane penetrative Ir-cR8-treated cancer cells manifested a slow onset of oncotic cell death featuring plasma membrane leakage and cell content eruption, which further exhibited dual potential in overcoming traditional chemotherapy drug-resistance of cancer cells and in subsequently inducing an inflammatory response in dendritic cells and triggering anti-tumour immunity in mice. Thus, these membrane active immunogenic complexes are in agreement with the trending development of chemotherapeutic drugs as cytotoxic agents that are widely effective for drug-resistant/non-resistant cancer cells and as a key component for immune-synergetic therapy.

## Ethical statement

This study was performed in strict accordance with the policy published in China State Council Gazette Supplement (Aug 20, 2017) “Regulations on Administration of Animals Used as Subjects of Experiments” and was approved by the Institutional Animal Care and Use Committee of Suzhou Institute of Nano-Tech and Nano-Bionics, Chinese Academy of Sciences.

## Conflicts of interest

There are no conflicts to declare.

## Supplementary Material

SC-011-D0SC03975F-s001
