# Supplementary material for: Structure-tuned membrane active Ir-complexed oligoarginine overcomes cancer cell drug resistance and triggers immune responses in mice
Source: Chem Sci. 2020 Aug 10;11(34):9126–33. doi: 10.1039/d0sc03975f (PMC8161536; doi:10.1039/d0sc03975f)
Supplement: SC-011-D0SC03975F-s001 [file SC-011-D0SC03975F-s001.pdf]

Electronic Supplementary Material (ESI) for Chemical Science.
This journal is © The Royal Society of Chemistry 2020

Structure-tuned Membrane Active Ir-complexed Oligoarginine Overcomes Cancer Cell Drug Resistance and Triggers Immune Responses in Mice

Shuangshuang Ji[a,b], Xiuzhu Yang[b], Xiaolong Chen[a,b], Ang Li[a,b], Doudou Yan[c], Haiyan Xu[c], Hao Fei[a,b*]

[a] School of Nano-Tech and Nano-Bionics, University of Science and Technology of China, Hefei, 230026, P R China.

[b] CAS Key Laboratory of Nano-Bio Interface, Division of Nanobiomedicine, Suzhou Institute of Nano-Tech and Nano-Bionics, Chinese Academy of Sciences, Suzhou, 215123, P R China.

[c] Institute of Basic Medical Sciences, Chinese Academy of Medical Sciences & Peking Union Medical College, Beijing, 100005, P R China.

Supplementary Information

Table of Contents

Materials and Methods

Table S1. Peptide sequences of RL1 and RL2--S5

Fig S1. Coordination mode of Ir-RL1/Ir-RL2---S6

Fig S2. Emission spectra of Ir-peptides, Ir-RL1, Ir-RL2 and [Ir(ppy)$_2$(H$_2$O)$_2$]OTf-----------S6

Fig S3-31. UPLC profiles and mass spectra of Ir-peptides, Ir-RL1, Ir-RL2------------S7

Fig S32. CD spectra of Ir-peptides---S37

Fig S33. Emission spectra of enantiomers ---S37

Fig S34. Cytotoxicity assay of [Ir(ppy)$_2$(H$_2$O)$_2$]OTf--S37

Fig S35. ROS generation assay of Ir-peptides, Ir-RL2, cR8--S38

Fig S36. Standard curves of Ir-peptides--S38

Fig S37. Time-series microscopic observation of HeLa cells treated with Ir-aR8 -------------S39

Fig S38. Endocytosis assay and intracellular distribution of Ir-cR8 ------------------------------S39

Fig S39. ROS inhibition 24 h MTT assay of Ir-cR8---S40

Fig S40. Caspase-3 activity assay of Ir-cR*n*--S40

Fig S41. Pan-caspase inhibition short-time MTT assay of Ir-cR8----------------------------------S40

Fig S42. Pan-caspase inhibition 24 h MTT assay of Ir-cR8--------------------------------------S41

Fig S43. Necroptosis inhibition short-time MTT assay of Ir-cR8---------------------------------S41

Fig S44. Necroptosis inhibition 24 h MTT assay of Ir-cR8--------------------------------------S41

Fig S45. Ferroptosis inhibition short-time MTT assay of Ir-cR8---------------------------------------S42

Fig S46. Ferroptosis inhibition 24 h MTT assay of Ir-cR8---S42

Fig S47. Calpain inhibition 24 h MTT assay of Ir-cR8---S42

Fig S48. Pyroptosis inhibition short-time MTT assay of Ir-cR8-----------------------------------S43

Fig S49. Pyroptosis inhibition 24 h MTT assay of Ir-cR8---S43

Fig S50. MTT assay of 293T or HUVEC cells treated with Ir-cR8----------------------------------S43

Materials and Methods

Materials

Chemical reagents including octanol, dimethyl sulfoxide (DMSO), trifluoroacetic acid were purchased from Sinopharm Chemical Reagent Co.,Ltd. Acetonitrile was purchased from Macklin. 9,10-Anthracenediyl-bis(methylene)-dimalonic acid (ABDA) was purchased from APExBIO. Thiazolyl Blue (MTT) was purchased from Adamas-Beta. E64, ferrostatin-1, necrostatin-1 were purchased from MedChemExpress. Paclitaxel, cytarabine, doxorubicin HCl, CCK-8, Propidium iodide (PI), 3,4-dichloroisocoumarin (DIC) were purchased from Meilunbio. 1M Tris-HCl buffer, chlorpromazine, N-Acetyl-L-cysteine (NAC) and Z-VAD-FMK were purchased from Beyotime. Dulbecco's modified Eagle medium (DMEM), RPMI 1640 medium and fetal bovine serum were purchased from Gibco. Tyrosine was purchased from Sigma. LysoTracker Red DND-99, Mito-TrackerRed FM, ER-TrackerTM Red were purchased from Invitrogen. Iridium standard was purchased from Guobiao (Beijing) Testing & Certification Co., Ltd. $[Ir(ppy)_2(H_2O)_2]$ was the same complex used before.[1] Peptides were ordered from Genscript (Nanjing). Animals were purchased from Changzhou Cavens Lab Animal Co. Ltd. IL-6, TNF-α ELISA kits were purchased from Cloud-Clone Corp., IL12(p70) Elisa kit was purchased from BOSTER Biological Technology Co. Ltd. Anti-mouse CD11c-FITC, anti-mouse CD80-PE, anti-mouse CD86-APC were purchased from Biolegend. Murine granulocyte-macrophage colony-stimulating factor (GM-CSF) and IL-4 were purchased from PeproTech.

Coordination reaction to form Ir-peptides

$[Ir(ppy)_2(H_2O)_2]$ was dissolved in DMSO for storage and quantified by ICP-mass. For coordination purpose, the storage solution was diluted in 50 mM Tris-HCl. cR*n* and aR*n* were quantified by Nanodrop (ThermoFisher) and dispersed in 50 mM Tris-HCl at the same concentration as $[Ir(ppy)_2(H_2O)_2]$ solution. The two solutions were mixed in one tube with the ratio of 1:1 (v/v) and coordinated overnight at 37°C to obtain Ir-cR*n* and Ir-aR*n*. The reaction yields were all over 95% as determined by UPLC analysis. RL1 was coordinated with $[Ir(ppy)_2(H_2O)_2]$ at a ratio of 1:5 (v/v) and incubated for 1 h at 50°C to get Ir-RL1, RL2 was coordinated with $[Ir(ppy)_2(H_2O)_2]$ at a ratio of 1:5 (v/v) and incubated for 12 h at 25°C to get Ir-RL2. The redundant $[Ir(ppy)_2(H_2O)_2]$ was centrifuged and filtered by 220 nm filter.

Emission spectra of $[Ir(ppy)_2(H_2O)_2]$, Ir-peptides, Ir-RL1, Ir-RL2

The coordinated Ir-peptides, Ir-RL1, Ir-RL2 and $[Ir(ppy)_2(H_2O)_2]$ were diluted for 20.0 μM in 50 mM tris-HCl and excited by 328 nm to get the emission spectra by fluorescence spectrophotometer (F-4600, HITACHI), Ex Slit:5.0 nm, Em Slit:5.0 nm, 650 V.

Standard curves of Ir-peptides

The coordinated Ir-peptides were diluted for various concentrations (1, 5, 10, 25, 50 μM) in Dulbecco's modified Eagle medium (DMEM) and incubated at 37°C for 24 h, then the samples were excited by 328 nm to get the emission spectra, Ex Slit:5.0 nm, Em Slit:10.0 nm,700 V. The fluorescence intensity at 520 nm was calculated to establish the standard curves. (F-4600, HITACHI)

ROS analysis

9,10-Anthracenediyl-bis(methylene)-dimalonic acid (ABDA, 50 μM) was used to test the ROS generation ability of $[Ir(ppy)_2(H_2O)_2]$, cR8, Ir-cR8, Ir-RL2 in 50 mM tris-HCl solution at various concentrations including 0, 1, 5, 10, 20 μM. The samples were irradiated under the lighting of biosafety hood (44 mW/cm^2 as measured by illuminance meter) for 0, 1, 4, 10, 30 min. The emission spectrum of each group excited by 380 nm was obtained by fluorescence spectrophotometer and the decrement of intensity at 407 nm was detected. Ex Slit:5.0 nm, Em Slit:5.0 nm, 500 V. (F-4600, HITACHI)

Coordination efficiency and hydrophobicity of peptides

Peptides with or without coordination were filtered by 220 nm filter and transferred to autosampler vials and separated on an UPLC by ACQUITY UPLC C18 BEH 1.7 μm, 50 mm × 2.1 mm column using an Acquity UPLC system. (Waters Corp., USA) The gradient mobile phase consisted of acetonitrile (A) and H_2O (B) both containing 0.1% trifluoroacetic acid. A typical 15 min sample run consisted of 2 min of 5% solvent A and 95% solvent B followed by an increment of solvent A up to 90% for the remaining 6 min. Then the proportion was held for 1 min and changed to 5% solvent A and 95% solvent B in the next 4 min, and held for 2 min. The analytes were monitored by a photo diode array (PDA) detector set at 220 nm and 254 nm and the flow rate was set to 0.3 mL min^{-1}.

Separation of isomer

Ir-cR5 was filtered and transferred to an autosampler vial and separated on an ACQUITY UPLC BEH Shield RP18 1.7 μm, 100

mm × 2.1 mm column. (Waters Corp., USA) The gradient mobile phase consisted of acetonitrile (A) and H_2O (B) both containing 0.1% trifluoroacetic acid. A typical 15 min sample run consisted of 2 min of 20% solvent A and 80% solvent B followed by an increment of solvent A up to 23% for the remaining 2 min. Then the proportion of solvent A was increased to 45% in the following 8 min and decreased to 20% in the last 3 min. The analytes were monitored by a photo diode array (PDA) detector set at 220 nm and 254 nm and the flow rate was set to 0.3 mL min^{-1}. The separated metallopeptides were collected to lyophilized and quantified by ICP-MASS, then for MTT assay.

Circular dichroism assay of Ir-peptides

Ir-aRn (n=3, 5, 7), Ir-cR3 and Ir-cR7 were collected by the UPLC method described above to remove DMSO. The collected Ir-peptides were lyophilized and quantified by ICP-MASS. The Ir-peptides, isomers, mixture of two isomers were diluted to same concentration for circular dichroism test. (Chirascan-plus, Applied photophysics)

Log*P* assay

Water and octanol (1:1, v/v) were saturated for 24 h at room temperature and separated as aqueous phase and oil phase. Ir-cR*n* or Ir-aR*n* were added in the tube containing 1 mL aqueous phase and 1 mL oil phase with a final concentration of 20 μM, Ir-cR*n* or Ir-aR*n* in 2 mL aqueous phase (40 μM) were used to establish standard curves. Samples were stirred for 24 h at room temperature, and groups only containing aqueous phase were diluted to various concentrations (1, 5, 10, 20, 40 μM) to build the standard curves by fluorescence spectrophotometer, Ex Slit:5.0 nm, Em Slit:5.0 nm, 650 V. Then the aqueous phase's fluorescence intensities in samples containing both oil and aqueous phases were scanned, the concentration in aqueous phase of each Ir-peptide was calculated by standard curve, the Ir-peptide's initial concentration in aqueous phase minus 24 hour's concentration in aqueous phase to obtain the 24 hour's concentration in oil phase, $\log P=\log(c_{oil}/c_{aqueous})$.

Cell culture

A549, HeLa, cisplatin-resistant A549/DDP cells were incubated in Dulbecco's modified Eagle medium (DMEM) with 10% fetal bovine serum, HL60, Cytarabine-resistant HL60/Ara-C, doxorubicin-resistant HL60/ADR and Paclitaxel-resistant A549/Taxol cells were incubated in RPMI 1640 medium with 10% fetal bovine serum, 37 ^{o}C, 5%CO_2. A549/Taxol cell line was obtained from Pharmacology Department, College of Pharmaceutical Sciences, Soochow University. HL60/Ara-C and HL60/ADR cells were obtained from Institute of Hematology and Blood Diseases Hospital, Chinese Academy of Medical Sciences & Peking Union Medical College.

Induction of cisplatin resistant A549/DDP cells

A549 cells were incubated in Dulbecco's modified Eagle medium (DMEM) with 10% fetal bovine serum, 37°C, 5%CO_2 until entering the logarithmic growth period. Then the medium was replaced by DMEM medium containing 10% fetal bovine serum and 0.125 μM cisplatin. The cells were incubated with cisplatin for 24 h and the medium was removed and no cisplatin DMEM medium containing 10% fetal bovine serum was added until the next logarithmic growth period. The induction followed this cycle until the concentration of cisplatin reached 4 μM. The concentration of cisplatin was twice as much as before in each induction. Then the cells were incubated with DMEM containing 10% fetal bovine serum and 4 μM cisplatin for two months and medium containing cisplatin was replaced by no-cisplatin medium two weeks before experiment.

Cytotoxicity

HeLa cells were cultured in 96-well microplates, 8*10^3 cells per well overnight. Ir-peptides, Ir-RL1 and $[Ir(ppy)_2(H_2O)_2]$ of various concentrations (0, 2, 4, 8, 16, 32, 64, 128 μM) were incubated with HeLa cells for 24 h. Thiazolyl Blue (MTT) was added in each well 4 h before analysis. The absorbance at 490 nm of each well was obtained by microplate spectrophotometer to calculate the cell viability. The cell viabilities of A549, A549/DDP, A549/Taxol cells incubated with Ir-cR8 (24 h), cisplatin (48 h), paclitaxel (48 h) or 293T, HUVEC cells incubated with Ir-cR8 (24 h) were obtained by the same method. (Victor X4, PerkinElmer) HL60, HL60/ADR, HL60/Ara-C cells were seeded in 96-well microplates, 5*10^4 cells per well. Ir-cR8, doxorubicin, cytarabine of various concentrations were incubated with cells for 48 h (for Ir-cR8 was 24 h). CCK-8 of 20 μL was added in each well and incubated for 4 h, then the absorbance at 450 nm of each well was obtained by microplate spectrophotometer to calculate the cell viability. (Victor X4, PerkinElmer)

Time-lapse cytotoxicity

HeLa cells were cultured in 96-well microplates, 8*10^3 cells per well overnight. Ir-aR8 (7 μM), Ir-cR8 (7 μM), Ir-RL1 (5 μM) were incubated with HeLa cells for 0, 15, 30, 60, 1440 min. MTT was added in each well 4 h before analysis. The absorbance at 490 nm of each well was obtained by microplate spectrophotometer to calculate the cell viability. (Victor X4, PerkinElmer)

Cellular uptake of Ir-peptides

HeLa cells were cultured in 6-well microplates, 3*10^5 cells per well overnight. Ir-cR*n*, Ir-aR*n* at the concentration of 1/2 IC_{50} μM were incubated with HeLa cells for 24 hours. The fluorescence intensity at 520 nm of supernatant after 24 h incubation was measured. The uptake ratios were calculated based on the fluorescence standard curves established above.

Time-lapse microscopic observation

HeLa cells were cultured in glass-bottomed well overnight, 2*10^5 cells per well, and incubated with Ir-aR8, Ir-cR8, Ir-RL1 at the IC_{50} concentration and PI (10 μg mL^{-1}), then the cells were observed by confocal laser scanning microscope (CLSM) at forty-second intervals. (OLYMPUS, FV3000)

Time-series microscopic observation

HeLa cells were cultured in glass-bottomed well overnight, 2*10^5 cells per well, and incubated with Ir-aR8, Ir-cR8(10 μM) and PI (10 μg mL^{-1}), then the cells were observed by confocal laser scanning microscope (CLSM) at five-second intervals. (OLYMPUS, FV3000)

Subcellular colocalization of Ir-cR8

HeLa cells were cultured in glass-bottomed well overnight, 3*10^5 cells per well. After washing with PBS, organelles including lysosomes, mitochondria, endoplasmic reticulum were stained by LysoTracker Red DND-99, Mito-TrackerRed FM, ER-TrackerTM Red (the working concentrations follow the instructions) respectively for 30 min, then the dyes were replaced by DMEM containing 5 μM Ir-cR8 for another 10 min incubation before observed by CLSM. (OLYMPUS, FV3000)

Cellular ROS analysis

HeLa cells were cultured in 6-well microplate, 3*10^5 cells per well overnight. After washing, PBS, Ir-cR8 at its IC_{50} concentration were incubated with cells for 0.5, 1 h. CellRox Deep Red (Thermo Fisher, USA), a fluorogenic probe for measuring cellular oxidative stress in live or fixed cell was then added for another 30 min incubation (the working concentration follow the instruction). Next, cells were digested and washed three times by PBS. The obtained cells were subjected to flow cytometry analysis. (Accuri C6, Bio-Rad)

Endocytosis inhibitor Assays

HeLa cells were seeded in 6-well microplate overnight, 3*10^5 cells per well overnight. Endocytosis inhibitor chlorpromazine (5.0 μg mL^{-1}) was pre-incubated with cells for 1 h. Ir-cR8 (5 μM) was added in each well and incubated at 37°C for 2 h except the 4°C group which was placed at 4°C for 2 h. Then the fluorescence intensity at 520 nm of Ir-cR8 in supernatant of each group was scanned and calculated by the standard curves built above.

HeLa cells were cultured in glass-bottomed well for overnight, 3*10^5 cells per well. After washing with PBS, 250 μg mL^{-1} Rhb-Dextran and 5 μM Ir-cR8 were added in the well and incubated with Hela cells for 1 h, then the cells were observed by confocal laser scanning microscope (CLSM). (OLYMPUS, FV3000)

Hemolysis analysis

Erythrocytes 1.4*10^7 per well were incubated with Ir-aR*n*, Ir-cR*n* (*n*=4, 6, 8), RL1, RL2 of various concentrations for 2 h, water was used as positive control and isotonic phosphate buffered solution was used as negative control. Then the samples were shaked at the speed of 87 rpm under 37°C and centrifuged at 3000 rpm for 5 min, the absorbance at 405 nm of supernatant was obtained by microplate spectrophotometer (Victor X4, PerkinElmer).

ROS inhibition assay

NAC is an ROS inhibitor. Cells of the ROS inhibition group were pre-incubated with NAC (10 mM) for 1 h. Afterwards, PBS, Ir-cR8 (7, 10 μM) were added into the wells with or without pre-incubation of NAC for 1 h. Cell viability at 15, 30, 45, 60 min or 24 h of each group was calculated by MTT assay.

Calpain inhibition assay

E64 is an irreversible calpain inhibitor. Cells of the calpain inhibition group were pre-incubated with E64 (15 μM) for 1h.

Afterwards, PBS, Ir-cR8 (7, 10 μM) were added into the wells with or without pre-incubation of E64 for 1 h. Cell viability at 15, 30, 45, 60 min or 24 h of each group was calculated by MTT assay.

Caspase activity/inhibition analysis

Ir-cRn (n=4, 6, 8) were incubated with HeLa cells for 0.5, 1 h at their IC_{50} concentrations respectively. Cells incubated with DMEM or staurosporine (1 μM) for 4 h was used as negative or positive control. 100 μL Caspase-Glo® 3/7 reagent (Promega, USA) was added into each well and incubated for 2 h. luminescence（RLU）intensity of each well was analyzed by microplate spectrophotometer (Victor X4, PerkinElmer).

Z-VAD-FMK is an inhibitor for caspase family. PBS, Ir-cR8 (7, 10 μM) were added to the wells with or without pre-incubation of Z-VAD-FMK (50 μM) for 1 h. The incubation time for short period MTT assay were 15, 30, 45, 60 min, for long time period incubation was 24 h. MTT was added into each well after incubation. The absorbance at 490 nm at each well was analyzed by microplate spectrophotometer (Victor X4, PerkinElmer).

Pyroptosis inhibition assay

HeLa cells (8*10^3 per well) were seeded in 96-well microplate for 12 h. For the granzyme A mediated pyroptosis inhibition groups, 3,4-dichloroisocoumarin (DIC, 10 μM) was pre-incubated with HeLa cells for 1 h. Then, PBS, Ir-cR8 (7, 10 μM) were added in wells with/without DIC and incubated for short or long period as mentioned above. Afterwards, MTT was added for 4 h incubation and then medium were replaced by DMSO. The absorbance at 490 nm was scanned by microplate spectrophotometer (Victor X4, PerkinElmer).

Ferroptosis inhibition assay

HeLa cells (8*10^3 per well) were seeded in 96-well microplate for 12 h. For the ferroptosis inhibition groups, ferrostatin-1 (5 μM) was pre-incubated with HeLa cells for 1 h. Then, PBS, Ir-cR8 (7, 10 μM) were added in wells with/without Ferrostatin-1 and incubated for short or long period as mentioned above. Afterwards, MTT was added for 4 h incubation and then medium were replaced by DMSO. The absorbance at 490 nm was scanned by microplate spectrophotometer (Victor X4, PerkinElmer).

Necroptosis inhibition assay

HeLa cells (8*10^3 per well) were seeded in 96-well microplate for 12 h. For the necroptosis inhibition groups, necrostatin-1 (40 μM) was pre-incubated with HeLa cells for 1 h. Then, PBS, Ir-cR8 (7, 10 μM) were added in wells with/without Necrostatin-1 and incubated for short or long period as mentioned above. Afterwards, MTT were added for 4 h incubation and then medium were replaced by DMSO. The absorbance at 490 nm was scanned by microplate spectrophotometer (Victor X4, PerkinElmer).

In vitro DC maturation

Dendritic cells were isolated from the bone marrow of 6-week-old BALB/c mice purchased from Changzhou Cavens Lab Animal Co. Ltd. and incubated with DMEM medium containing 10% FBS, 100U/mL penicillin-streptomycin, 20 ng/mL murine granulocyte-macrophage colony-stimulating factor (GM-CSF) and 5 ng/ml IL-4. 4T1 cells were incubated with PBS, Ir-cR8, cisplatin and the supernatant of each group was added into dendritic cells for 24 h incubation. The DC were collected and stained with anti-CD11c-FITC, anti-CD80-PE, anti-CD86-APC for flow cytometer analysis.

In vivo Cytokines assay

4T1 cells were implanted at left flank of 6-week-old BALB/c mice on day 0 and right flank on day 4. On day 7, Ir-cR8(0.1 μmol) or PBS was intra-tumor injected at left flank. On day 14, the tumors of right flank were collected and homogenized. The supernatant of each tumor was collected for ELISA analysis follow the kit's instruction.

Animal welfare

This study was performed in strict accordance with the policy published in China State Council Gazette Supplement (Aug 20, 2017) "Regulations on Administration of Animals Used as Subjects of Experiments" and was approved by the Institutional Animal Care and Use Committee of Suzhou Institute of Nano-Tech and Nano-Bionics, Chinese Academy of Sciences.

Figures and Tables

Table S1: Peptide sequences of RL1 and RL2

Peptide	N-terminus	Sequence	C-terminus
RL1	/	HGGRLLRLLRRLLRLLR	-amide
RL2	/	HGGRLLRLLRLRRLLRL	-amide

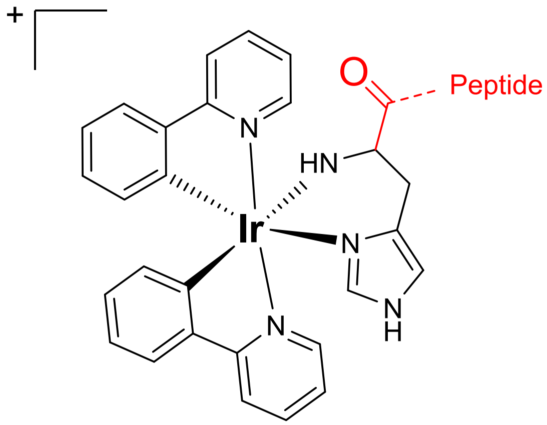

Fig S1. Coordination mode of RL1/RL2 with $[Ir(ppy)_2(H_2O)_2]$.

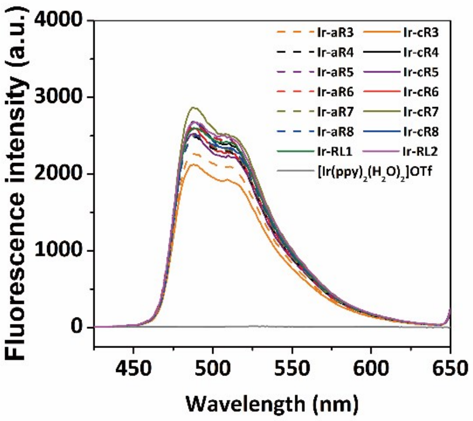

Fig S2. Emission spectra of Ir-peptides, Ir-RL1, Ir-RL2 and $[Ir(ppy)_2(H_2O)_2]OTf$.

aR3

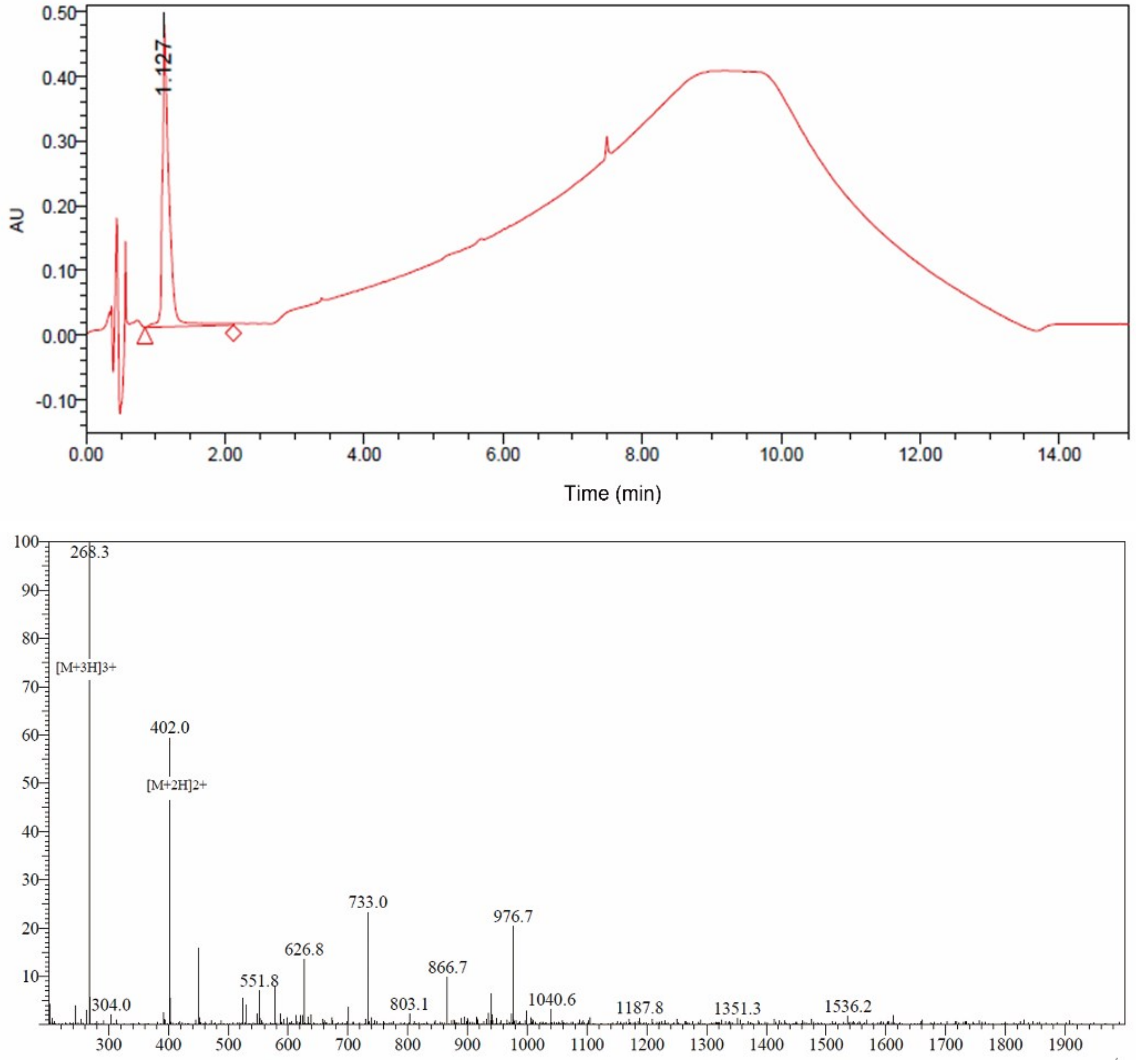

Theoretical MW: 801.91

Observed MW: 801.9

Fig S3. UPLC retention time (upper panel) and Mass-spectrum (lower panel) of aR3.

Ir-aR3

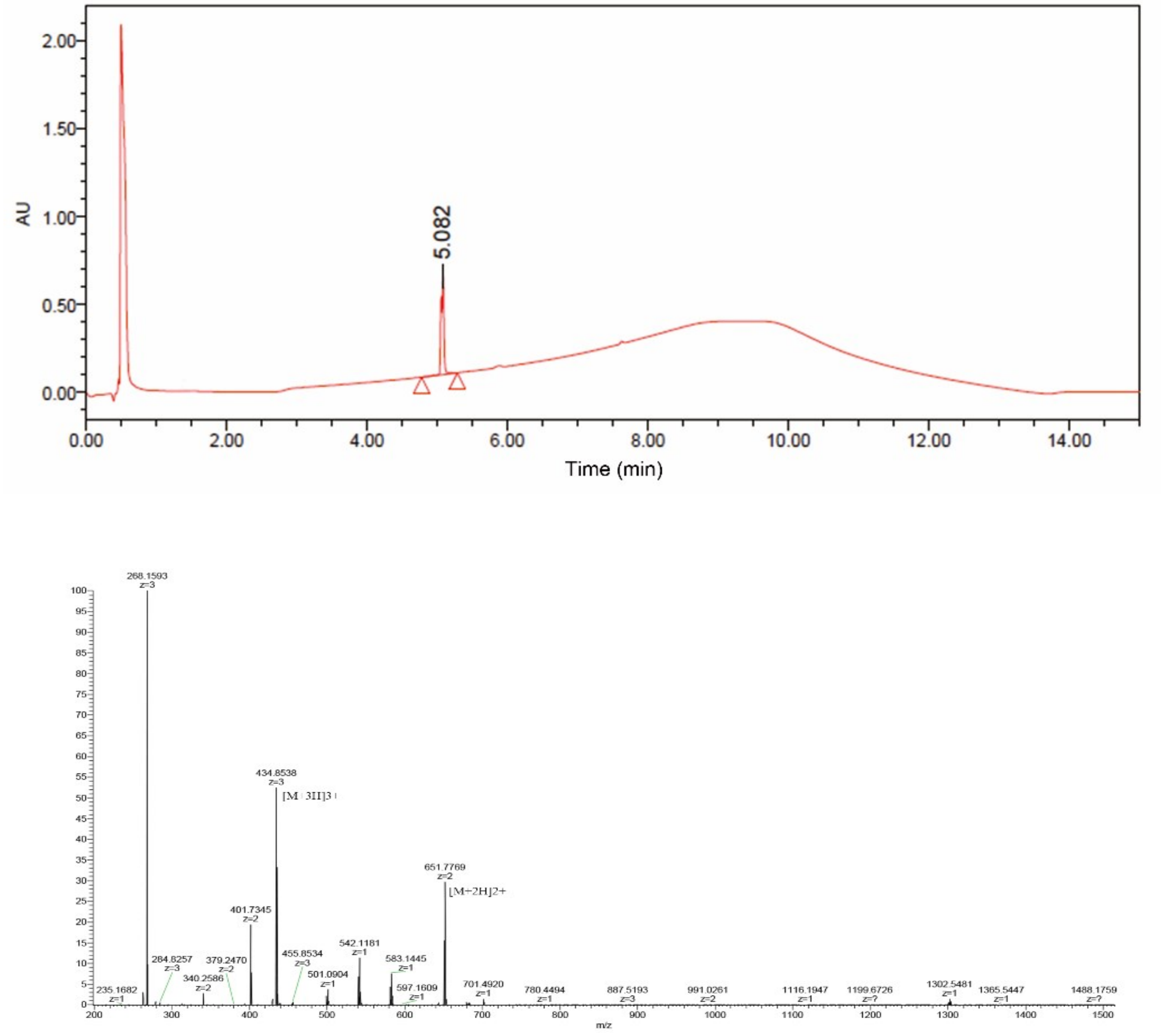

Theoretical MW: 1301.91

Observed MW: 1301.56

Fig S4. UPLC retention time (upper panel) and Mass-spectrum (lower panel) of Ir-aR3.

cR3

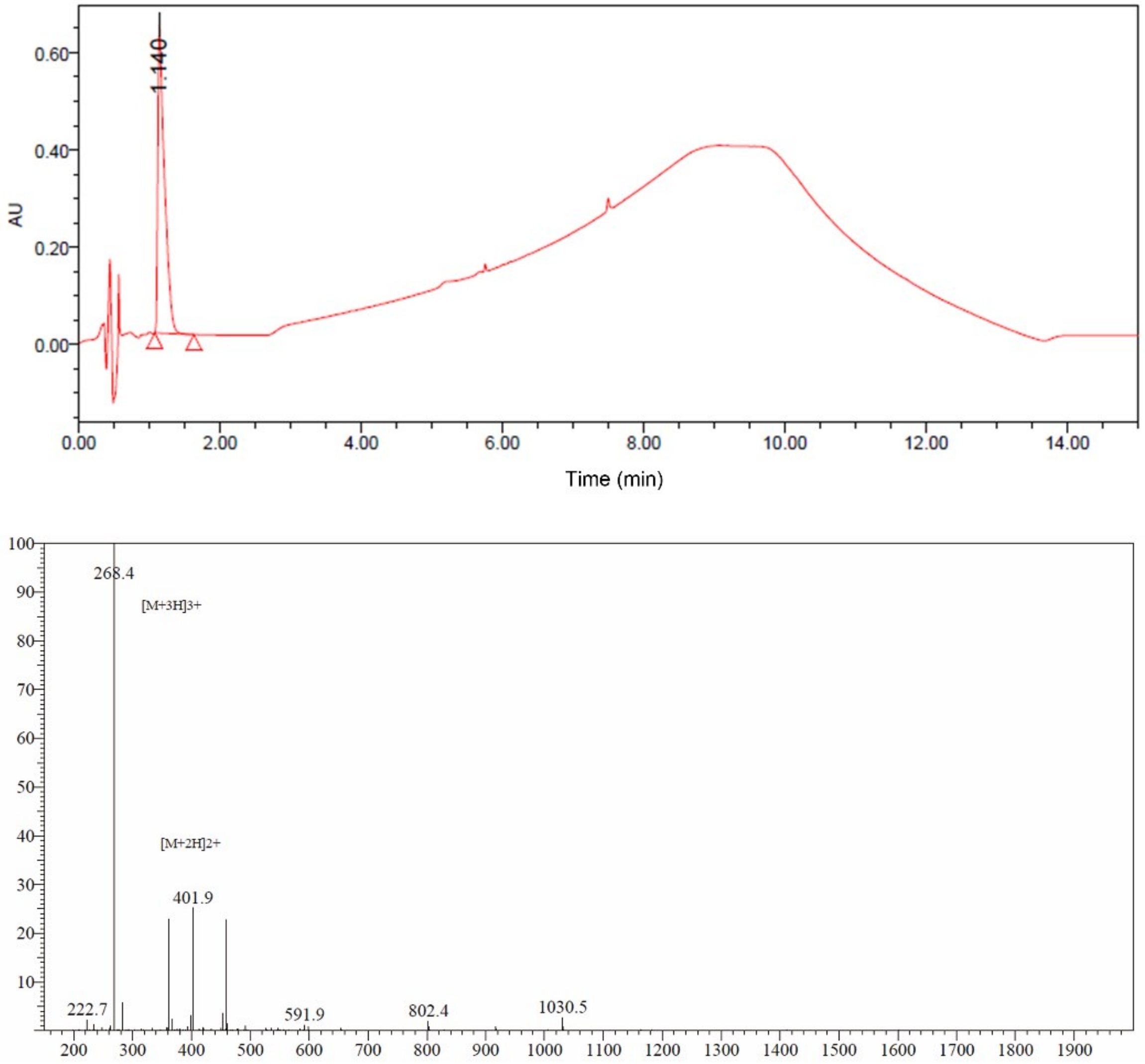

Theoretical MW: 801.91

Observed MW: 802.2

Fig S5. UPLC retention time (upper panel) and Mass-spectrum (lower panel) of cR3.

Ir-cR3

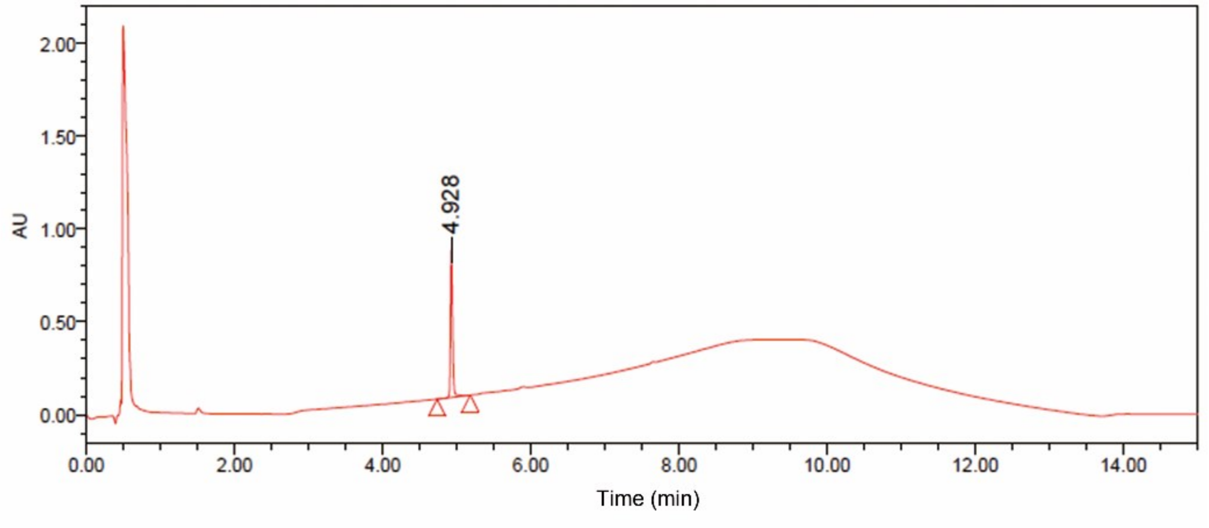

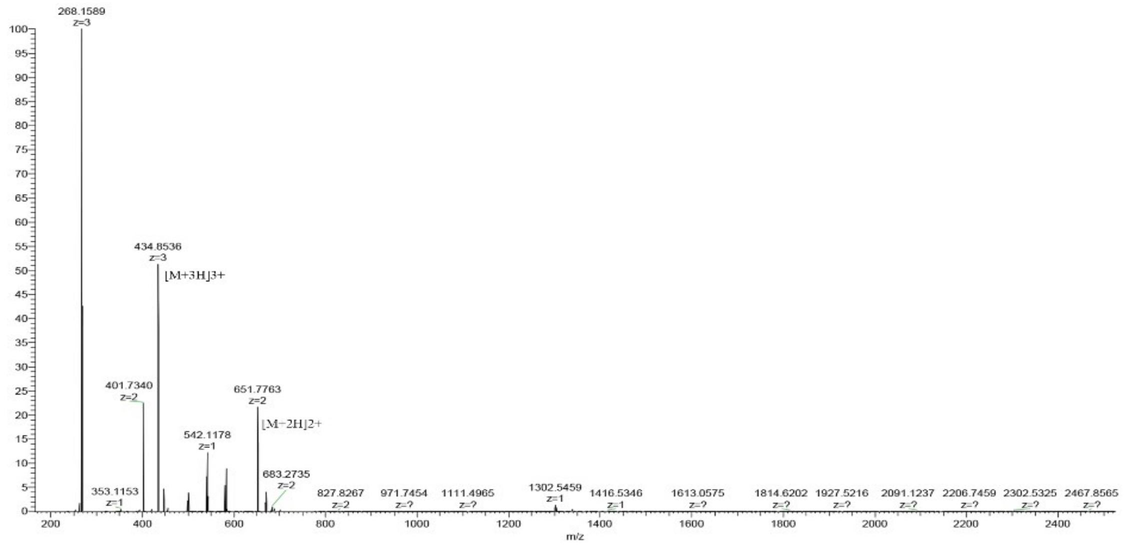

Theoretical MW: 1301.91

Observed MW: 1301.56

Fig S6. UPLC retention time (upper panel) and Mass-spectrum (lower panel) of Ir-cR3.

aR4

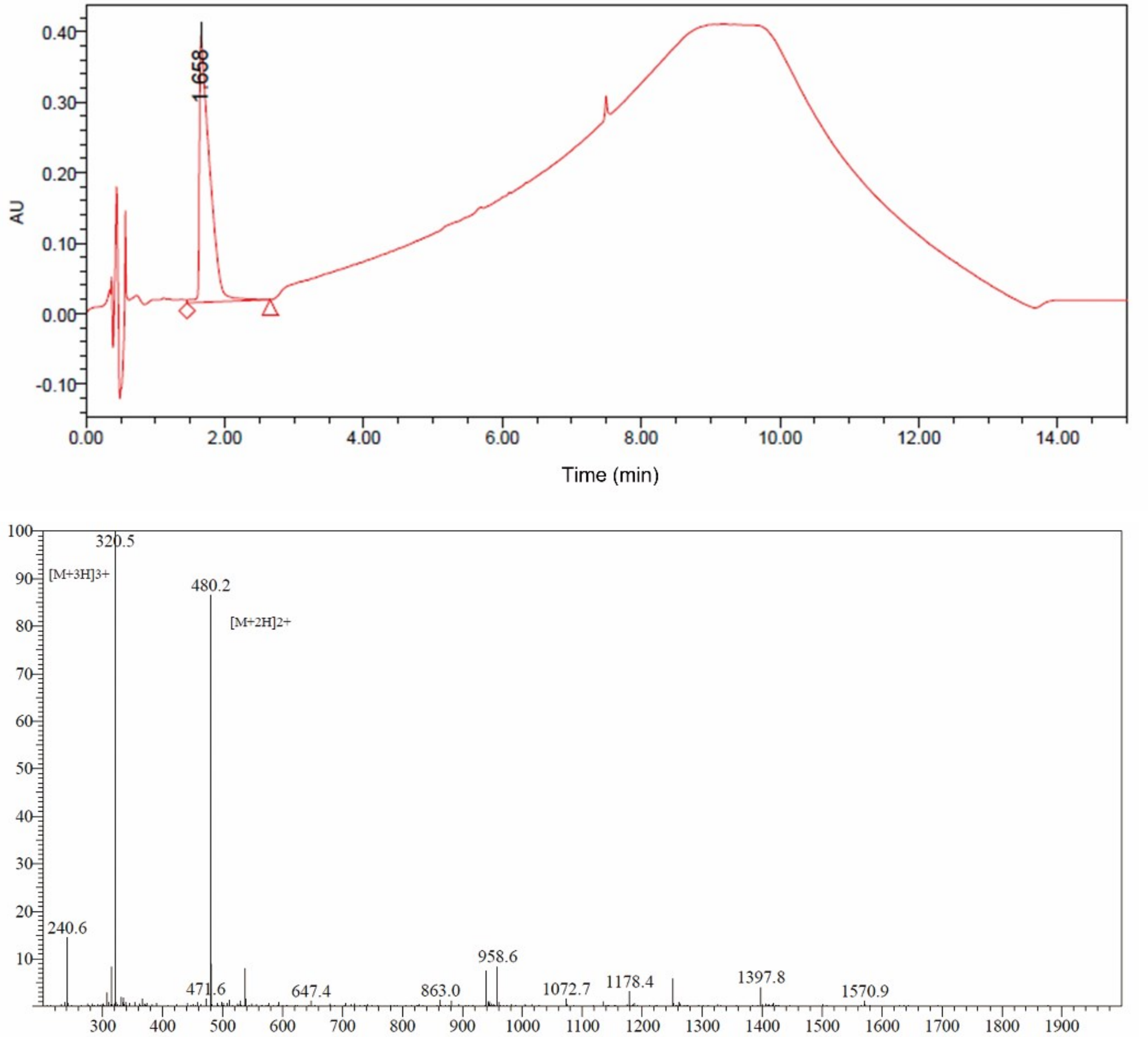

Theoretical MW: 958.09

Observed MW: 958.5

Fig S7. UPLC retention time (upper panel) and Mass-spectrum (lower panel) of aR4.

Ir-aR4

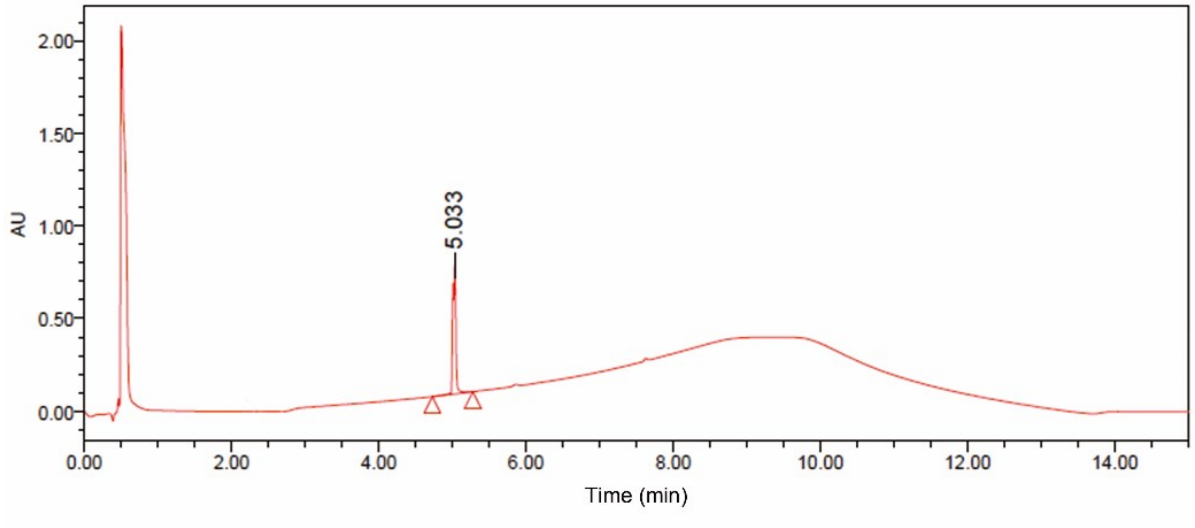

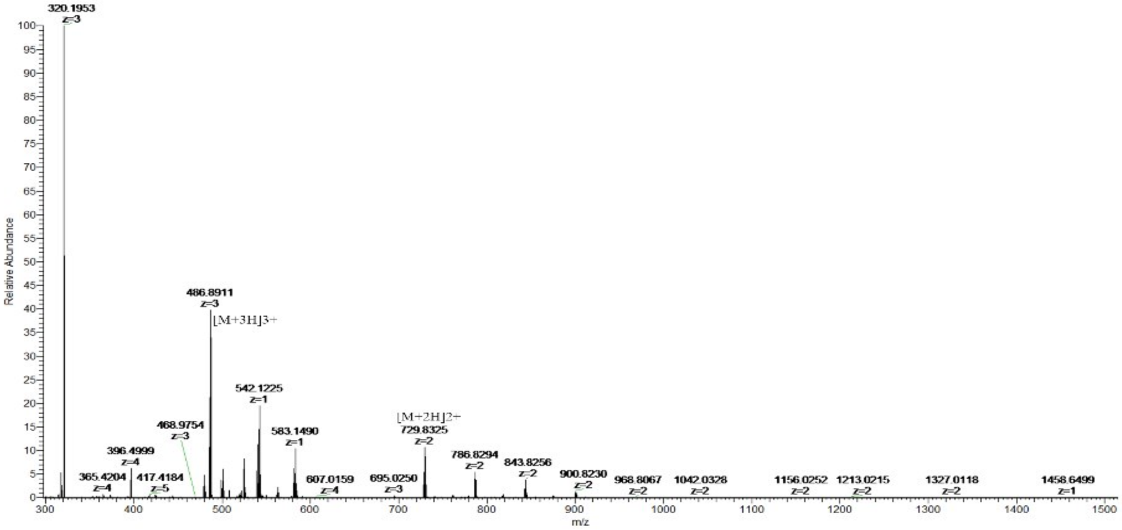

Theoretical MW: 1458.09

Observed MW: 1457.67

Fig S8. UPLC retention time (upper panel) and Mass-spectrum (lower panel) of Ir-aR4.

cR4

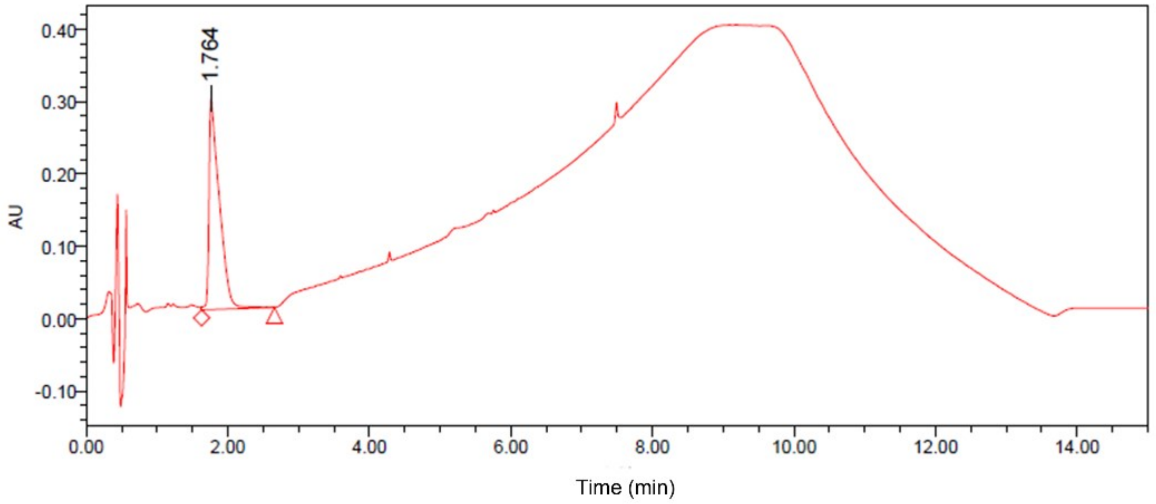

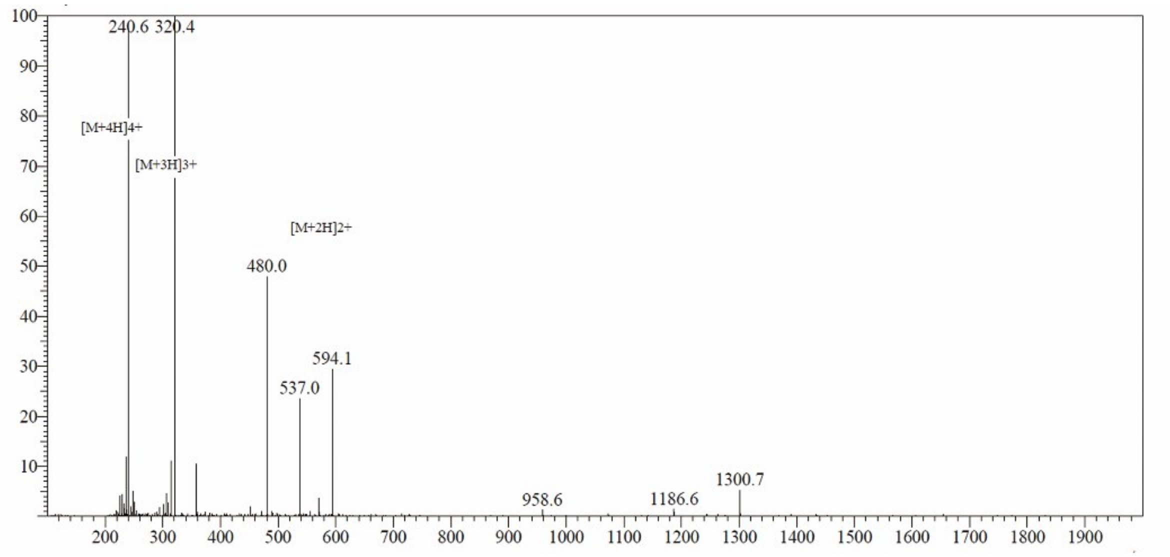

Theoretical MW: 958.09

Observed MW: 958.2

Fig S9. UPLC retention time (upper panel) and Mass-spectrum (lower panel) of cR4.

Ir-cR4

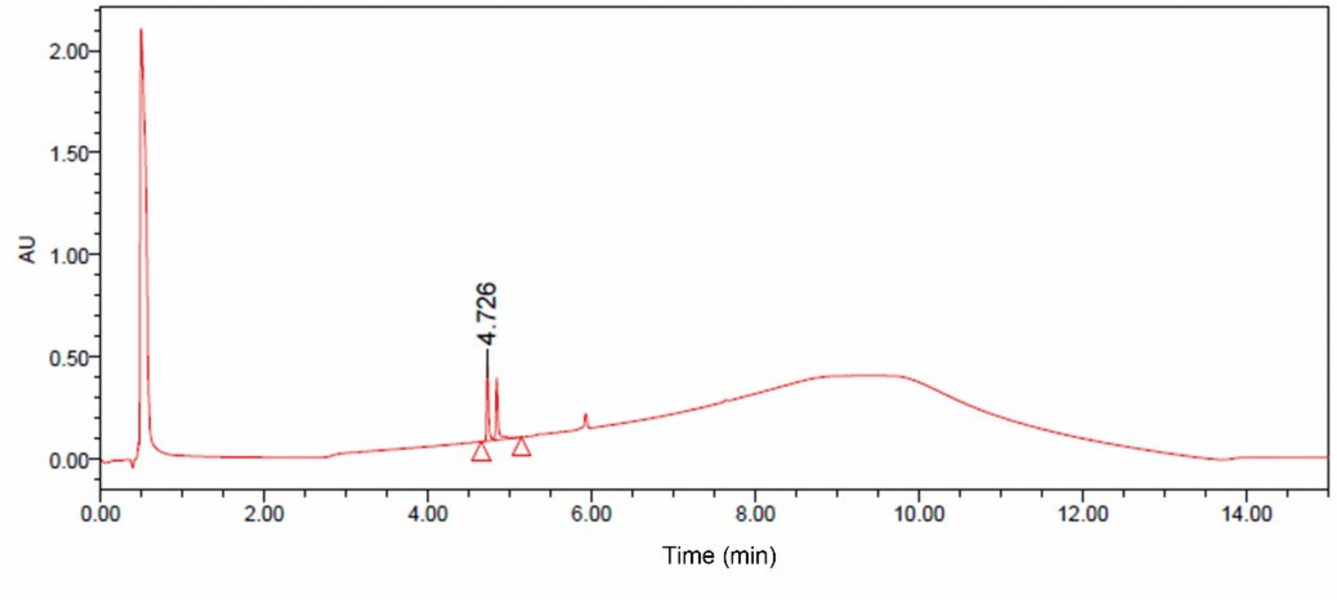

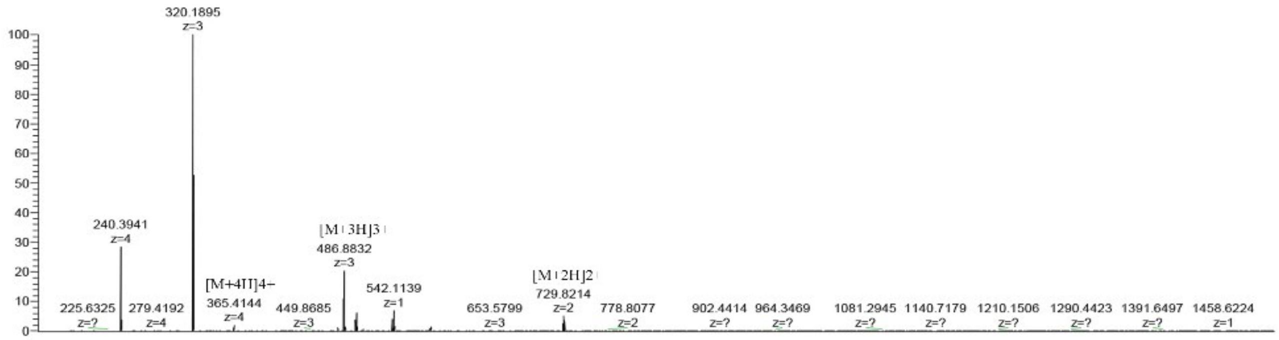

Theoretical MW: 1458.09

Observed MW: 1457.65

Fig S10. UPLC retention time (upper panel) and Mass-spectrum (lower panel) of Ir-cR4.

aR5

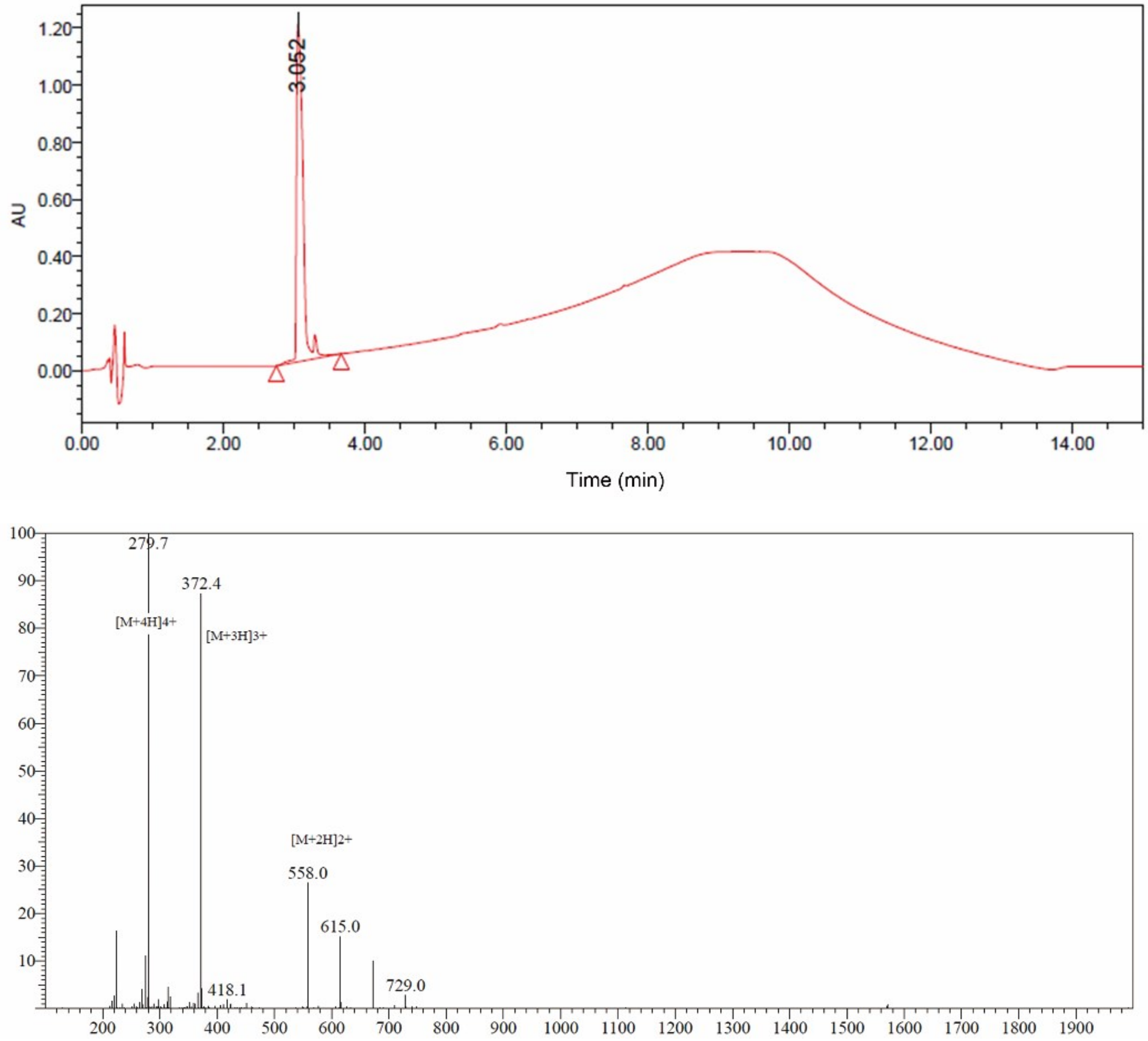

Theoretical MW: 1114.28

Observed MW: 1114.8

Fig S11. UPLC retention time (upper panel) and Mass-spectrum (lower panel) of aR5.

Ir-aR5

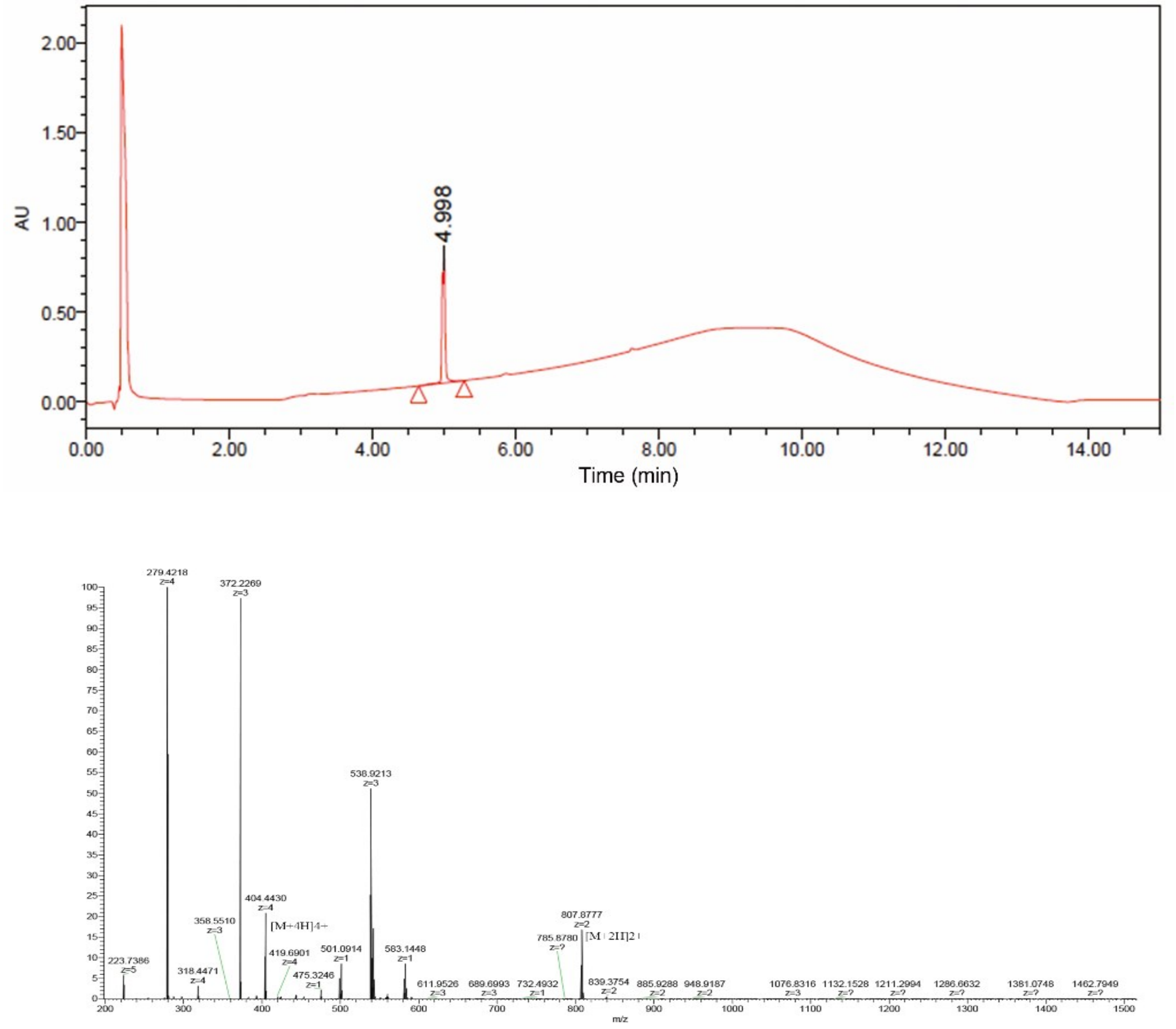

Theoretical MW: 1614.28

Observed MW: 1613.77

Fig S12. UPLC retention time (upper panel) and Mass-spectrum (lower panel) of Ir-aR5.

cR5

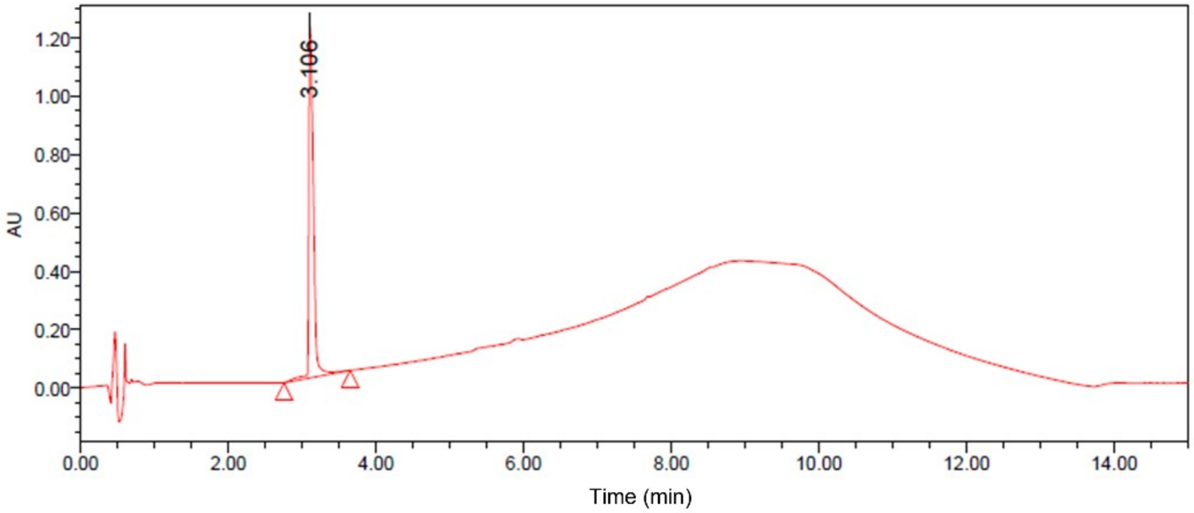

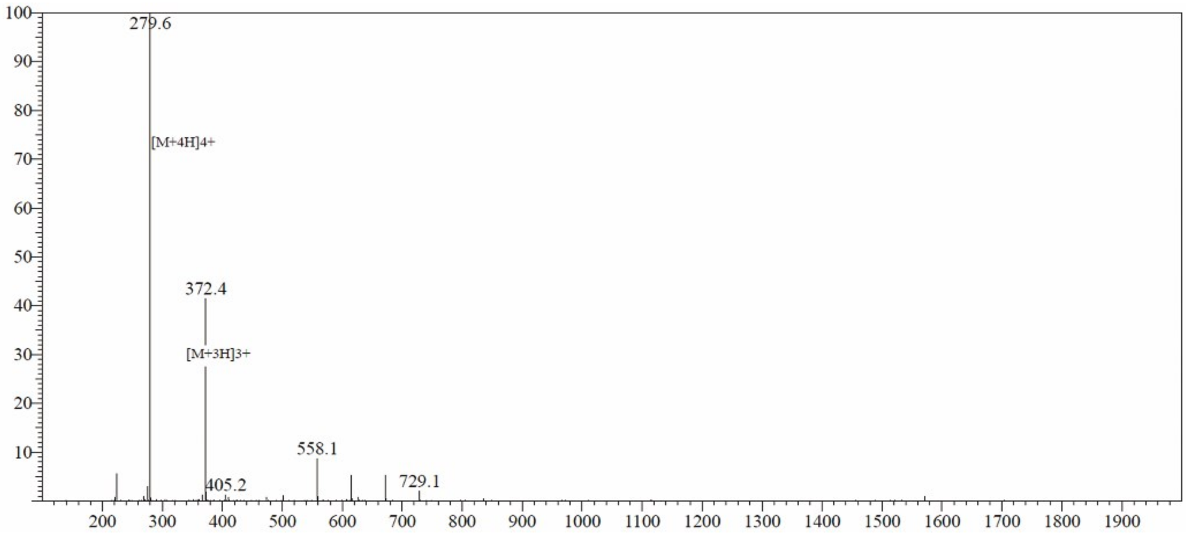

Theoretical MW: 1114.28

Observed MW: 1114.4

Fig S13. UPLC retention time (upper panel) and Mass-spectrum (lower panel) of cR5.

Ir-cR5

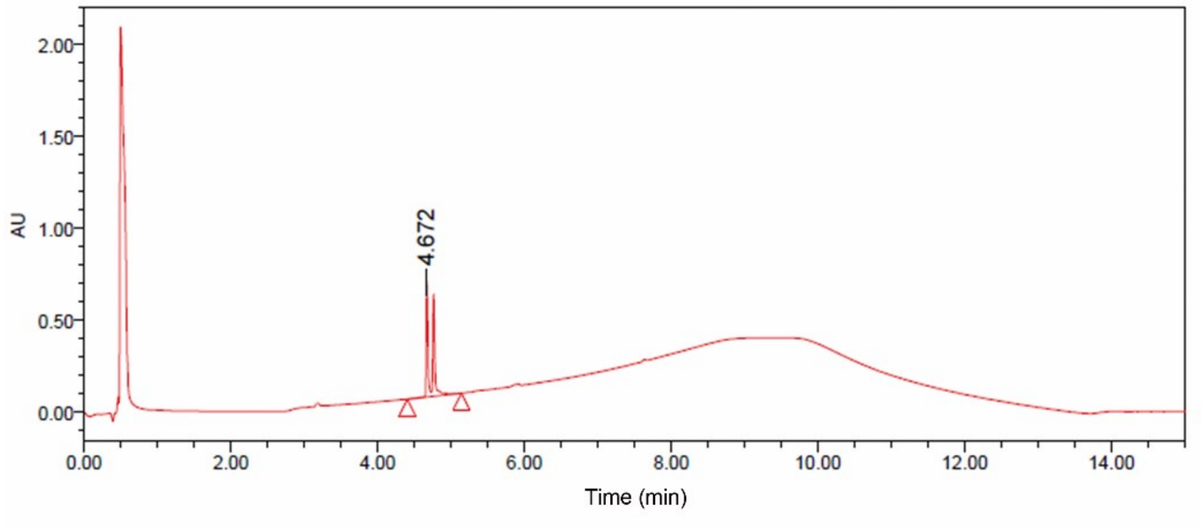

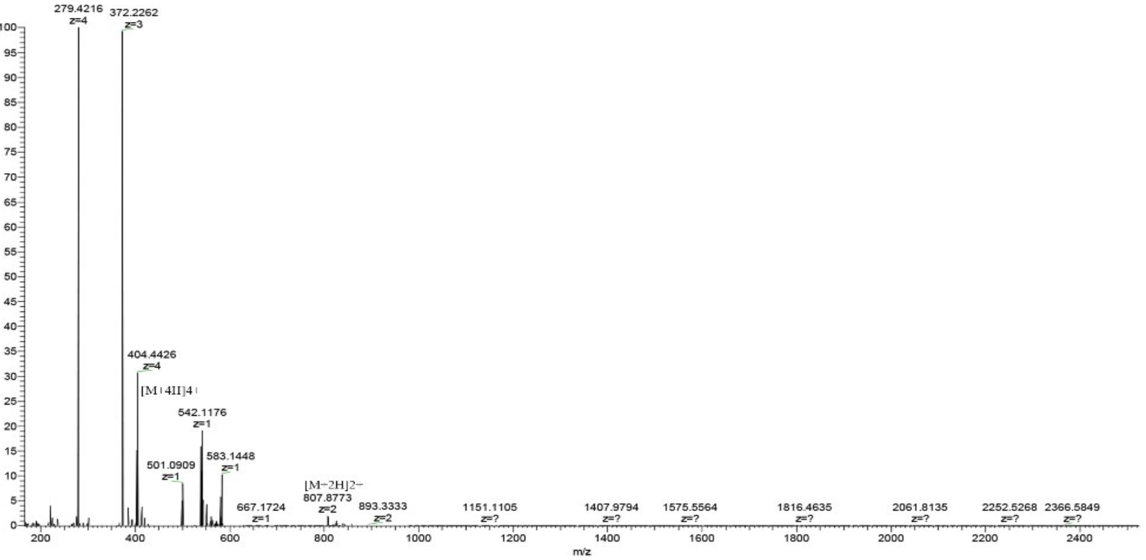

Theoretical MW: 1614.28

Observed MW: 1613.77

Fig S14. UPLC retention time (upper panel) and Mass-spectrum (lower panel) of Ir-cR5.

Isomer separation of Ir-cR5

A

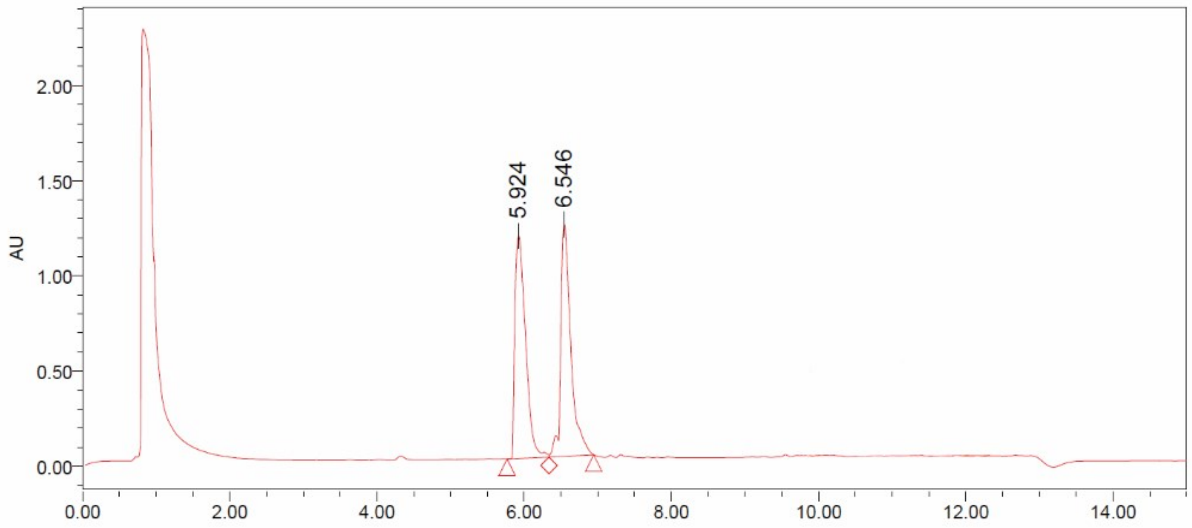

B

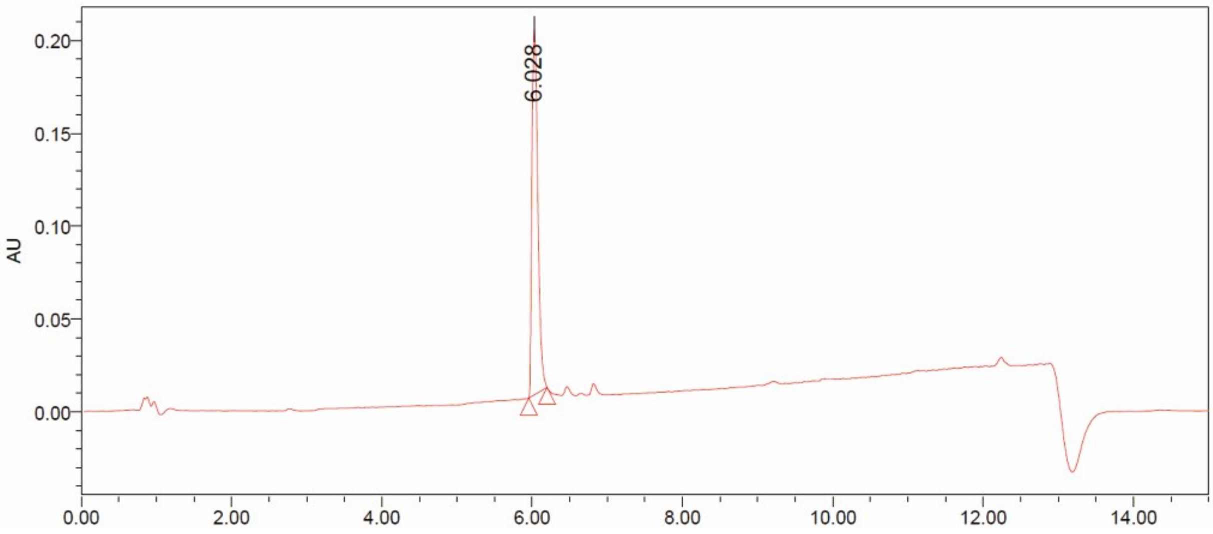

C

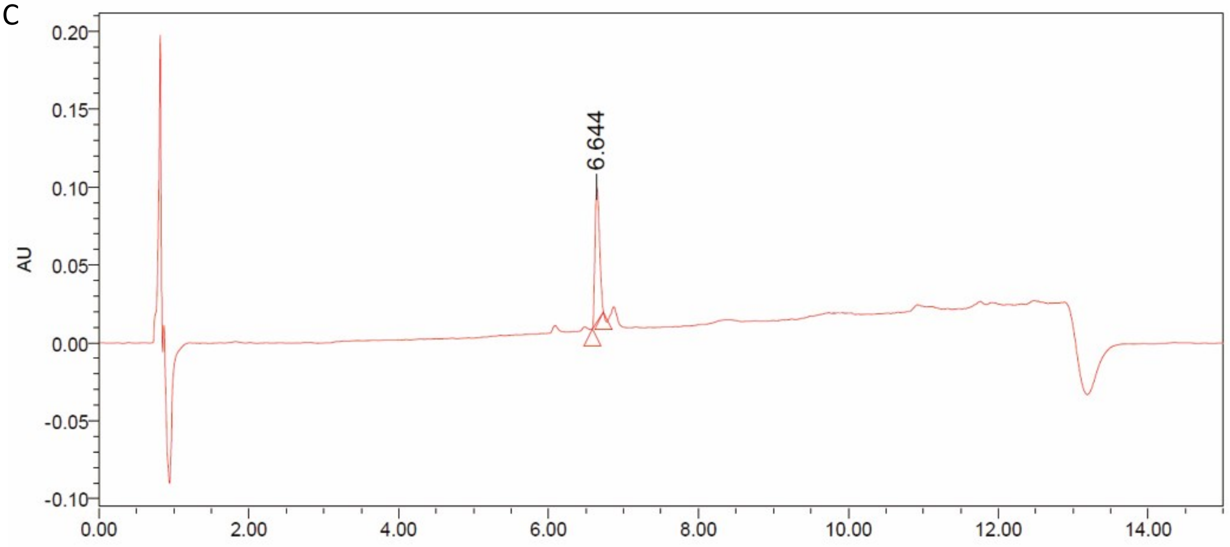

D

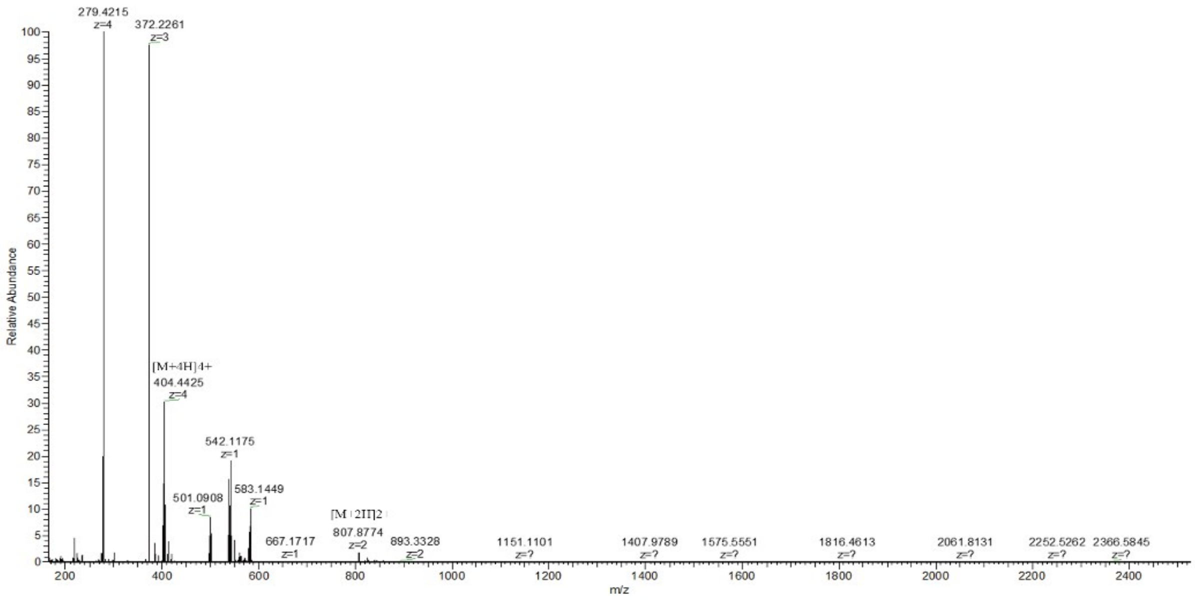

E

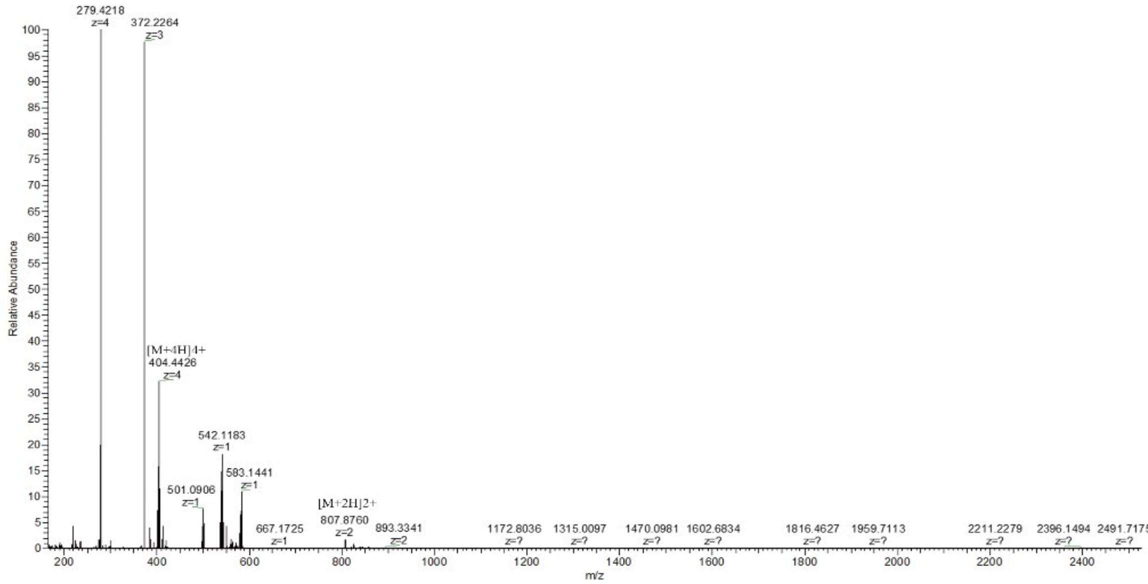

Fig S15. Ir-cR5 contains isomers(A) and the two ingredients were separated by UPLC (B, C), LC-MASS verified their molecular weights were the same (D, E), their IC_{50}s(24 h) against HeLa cells were 23.6, 24.3 μM respectively.

aR6

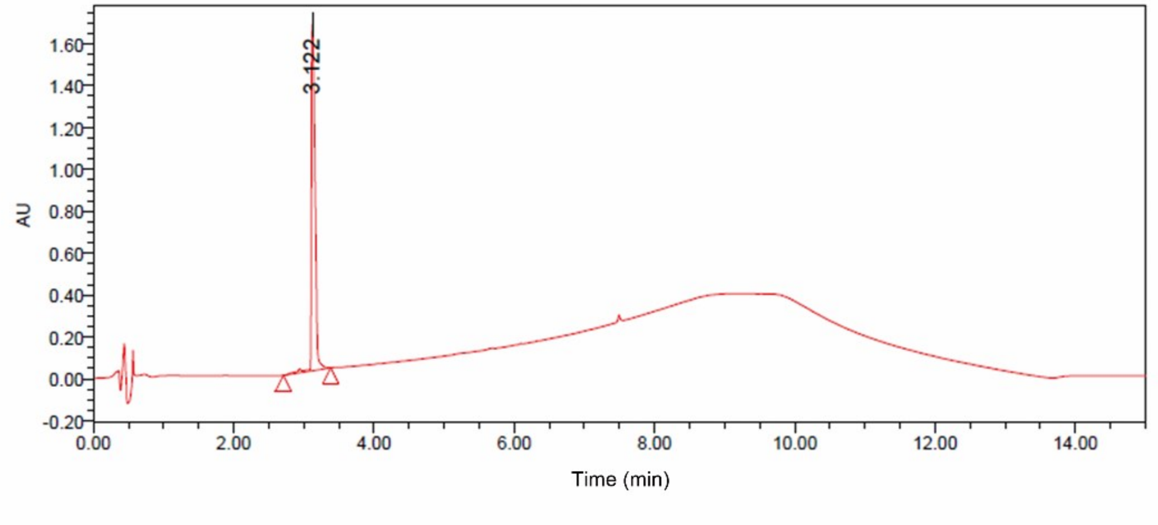

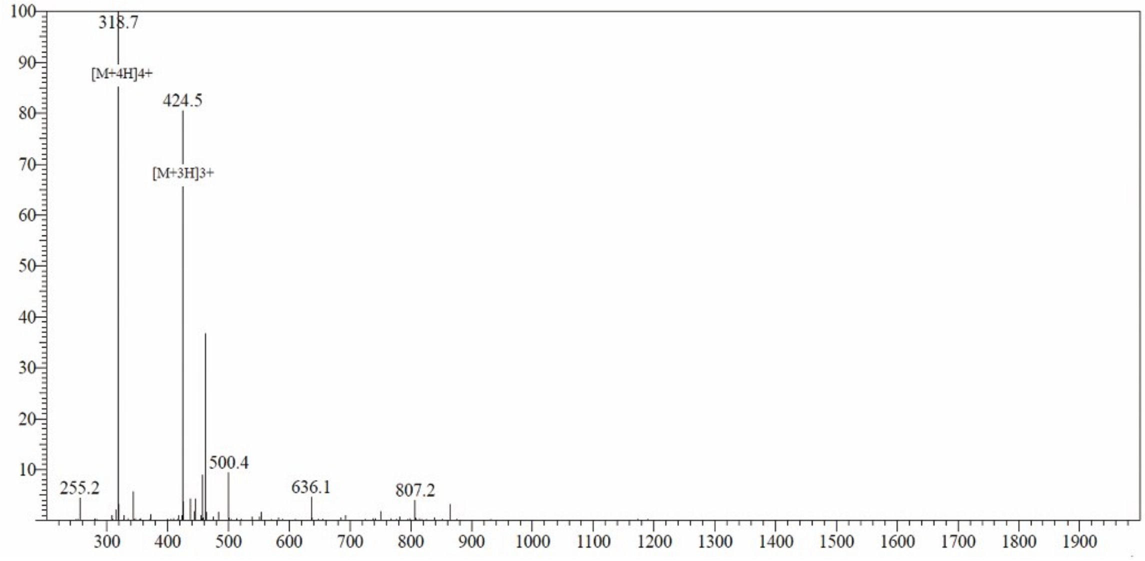

Theoretical MW: 1270.47

Observed MW: 1270.8

Fig S16. UPLC retention time (upper panel) and Mass-spectrum (lower panel) of aR6.

Ir-aR6

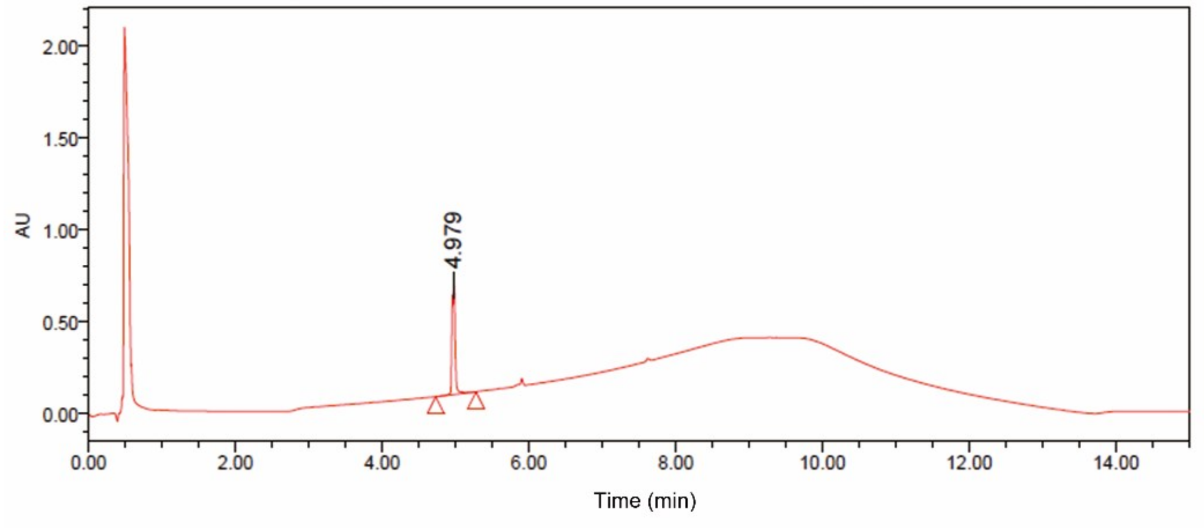

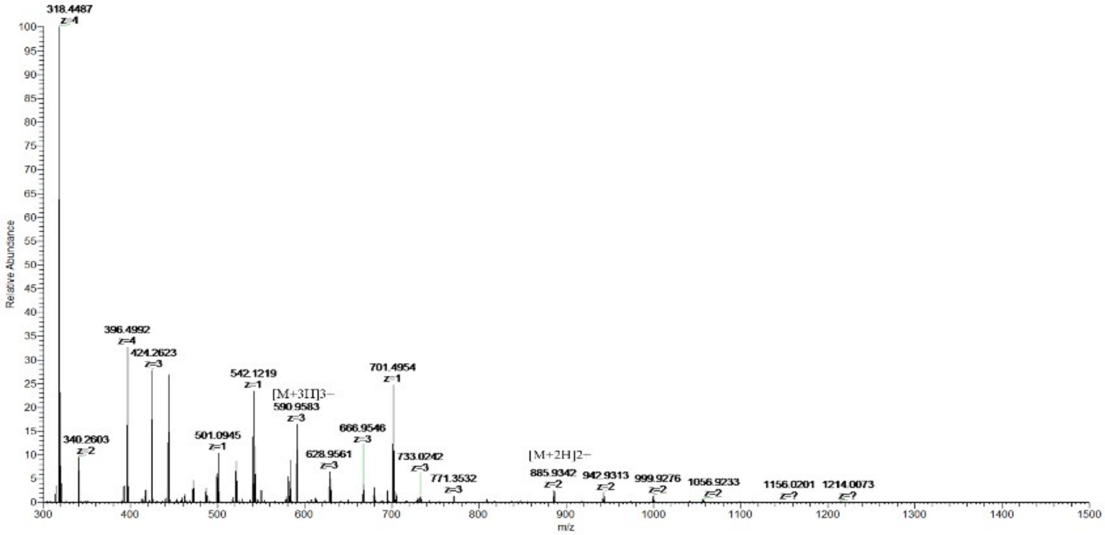

Theoretical MW: 1770.47

Observed MW: 1769.87

Fig S17. UPLC retention time (upper panel) and Mass-spectrum (lower panel) of Ir-aR6.

cR6

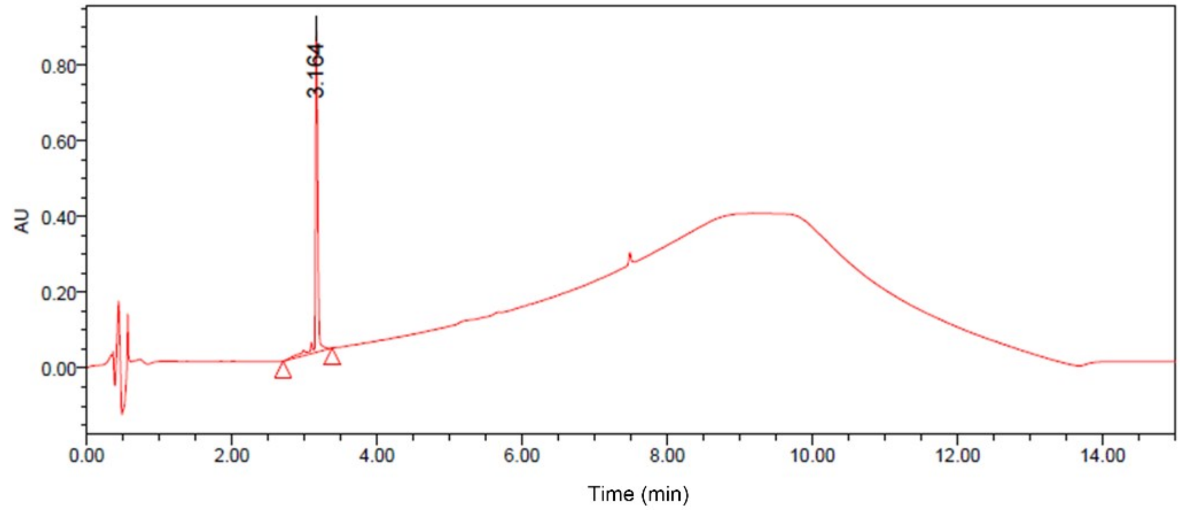

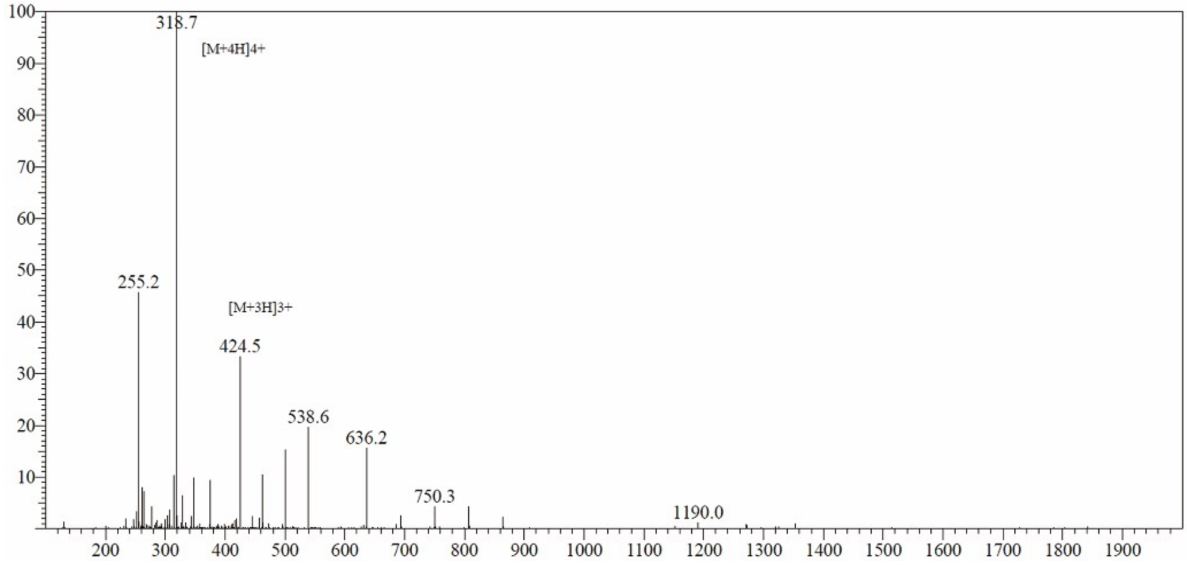

Theoretical MW: 1270.47

Observed MW: 1270.8

Fig S18. UPLC retention time (upper panel) and Mass-spectrum (lower panel) of cR6.

Ir-cR6

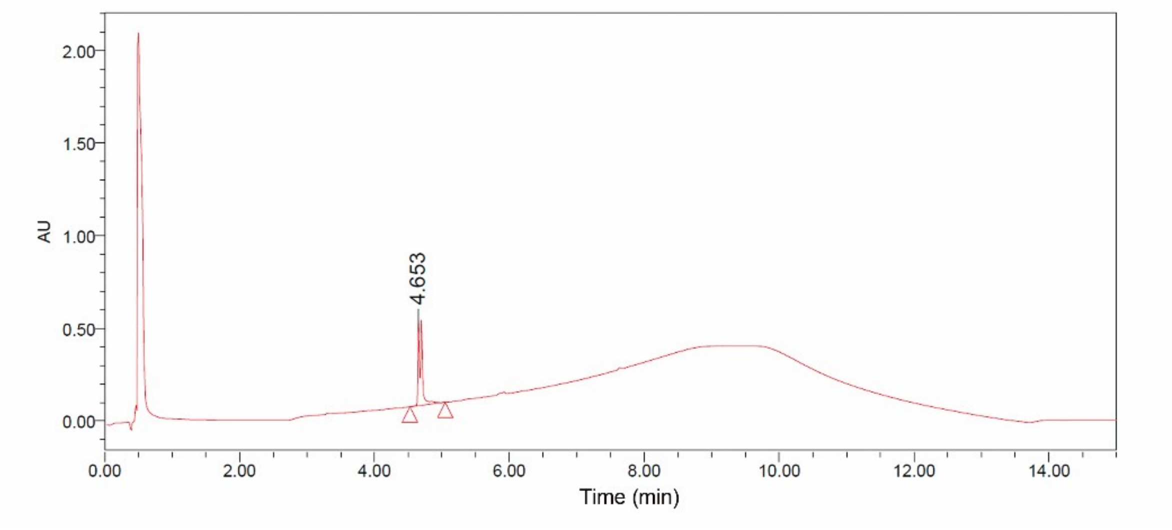

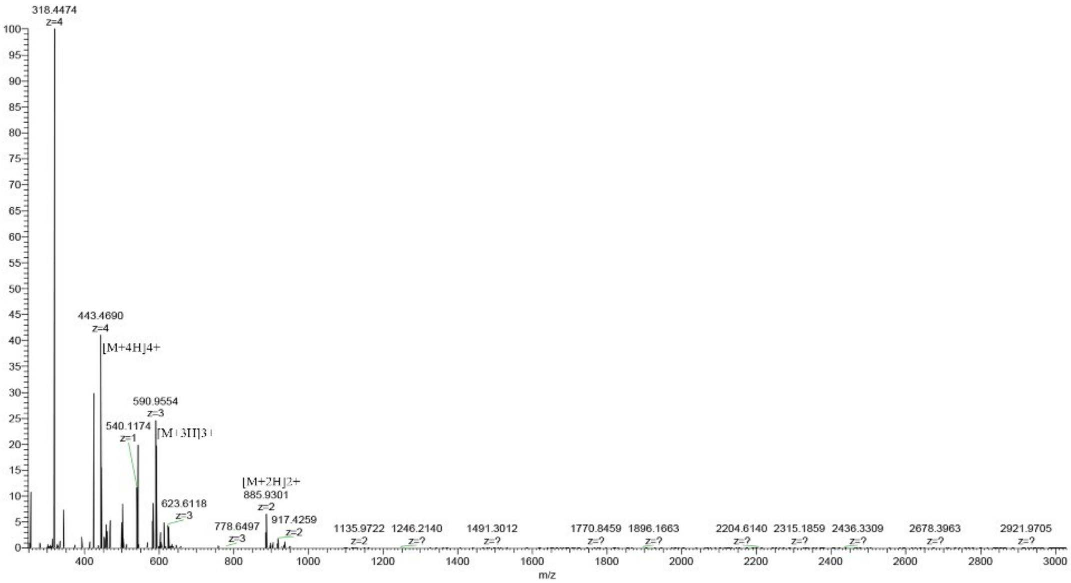

Theoretical MW: 1770.47

Observed MW: 1769.88

Fig S19. UPLC retention time (upper panel) and Mass-spectrum (lower panel) of Ir-cR6.

aR7

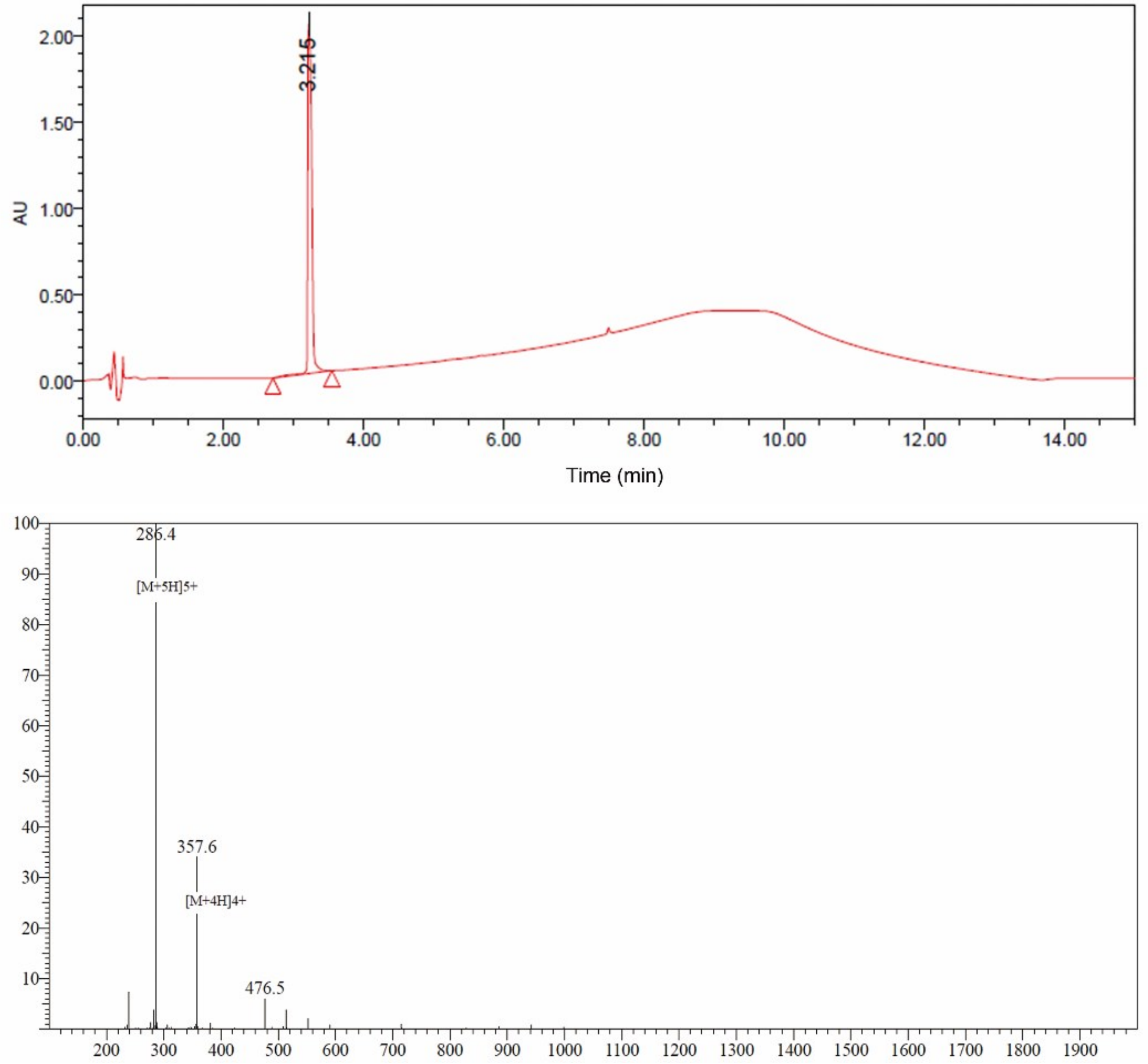

Theoretical MW: 1426.65

Observed MW: 1427

Fig S20. UPLC retention time (upper panel) and Mass-spectrum (lower panel) of aR7.

Ir-aR7

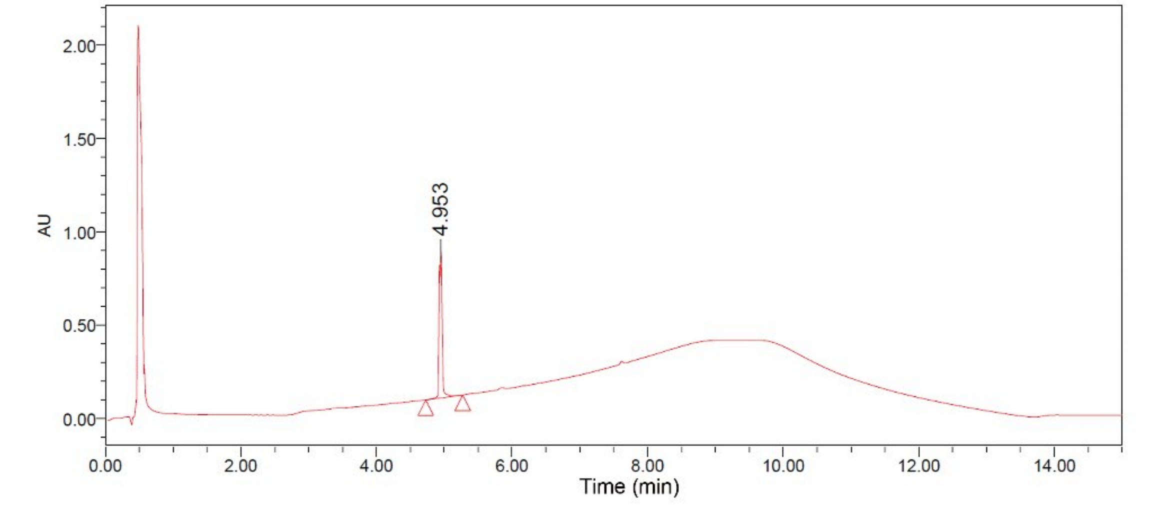

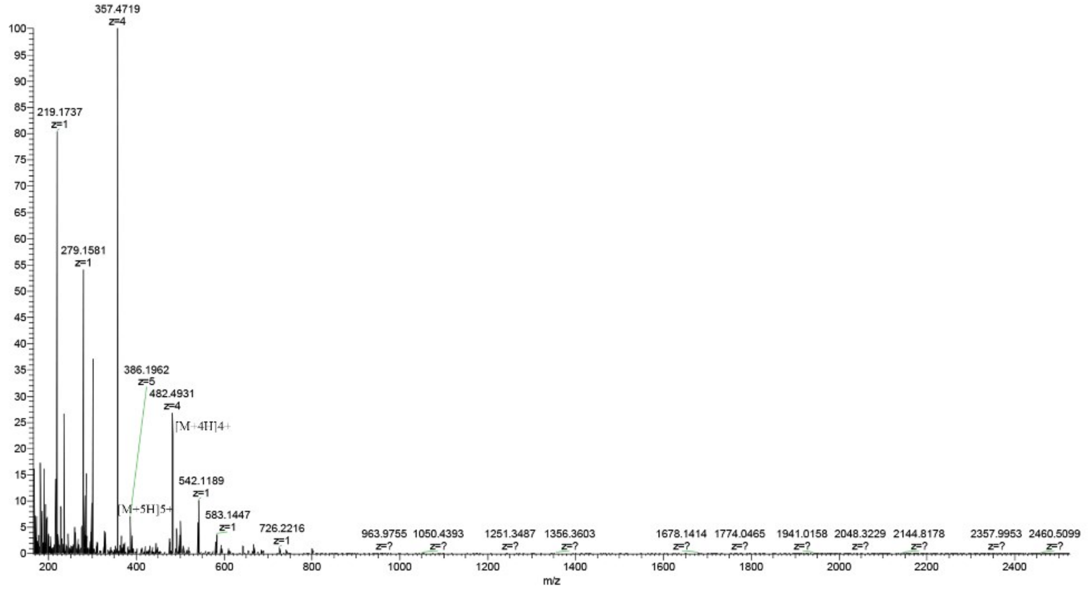

Theoretical MW: 1926.65

Observed MW: 1925.97

Fig S21. UPLC retention time (upper panel) and Mass-spectrum (lower panel) of Ir-aR7.

cR7

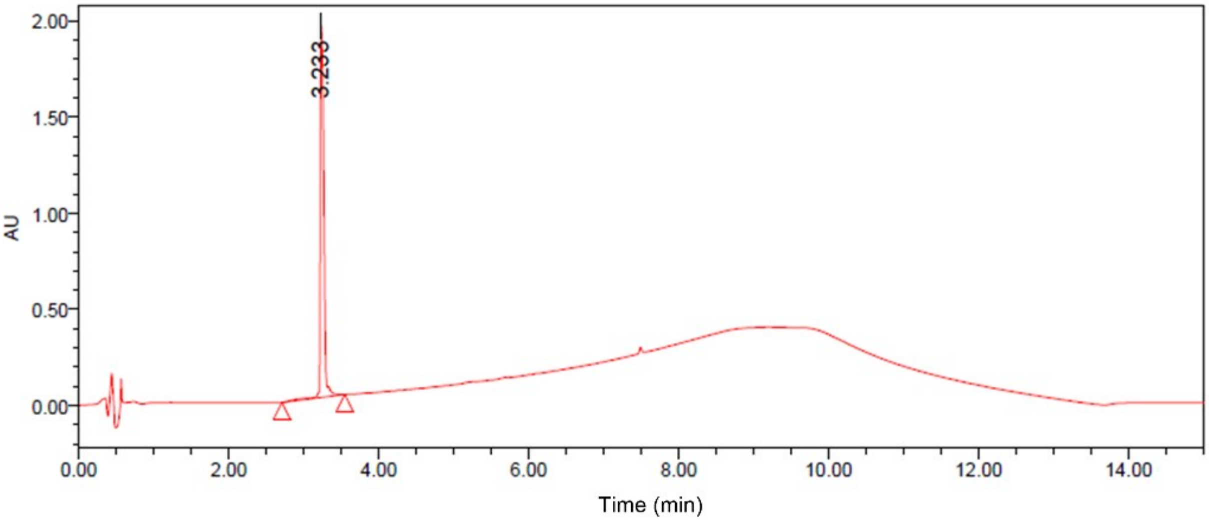

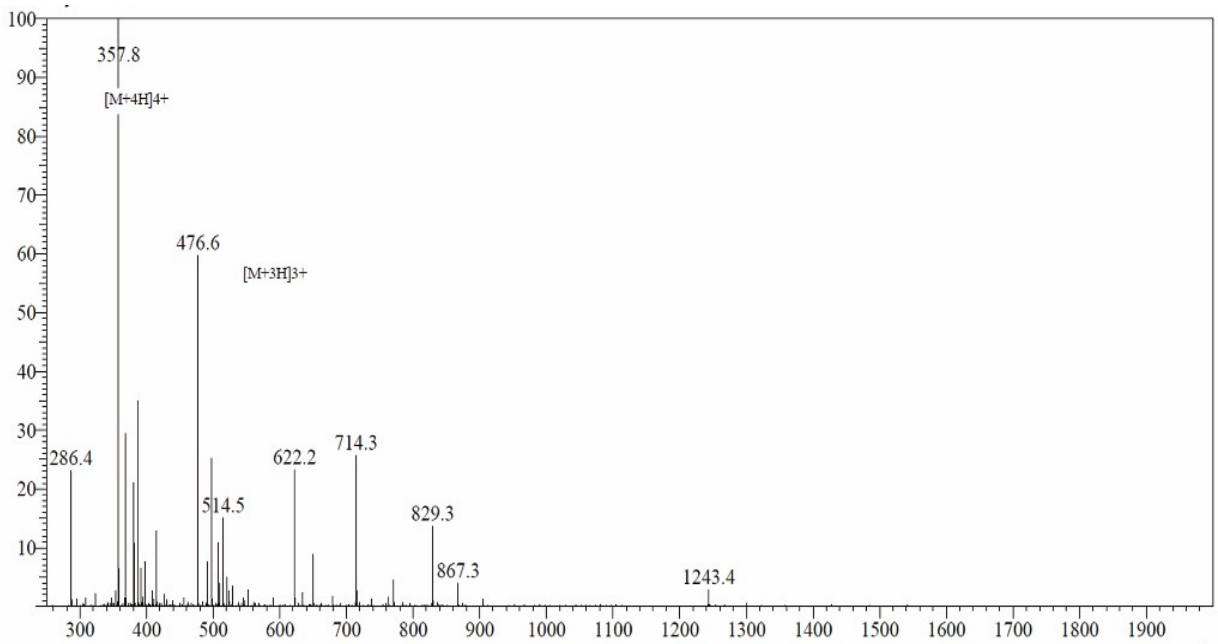

Theoretical MW: 1426.65

Observed MW: 1427

Fig S22. UPLC retention time (upper panel) and Mass-spectrum (lower panel) of cR7.

Ir-cR7

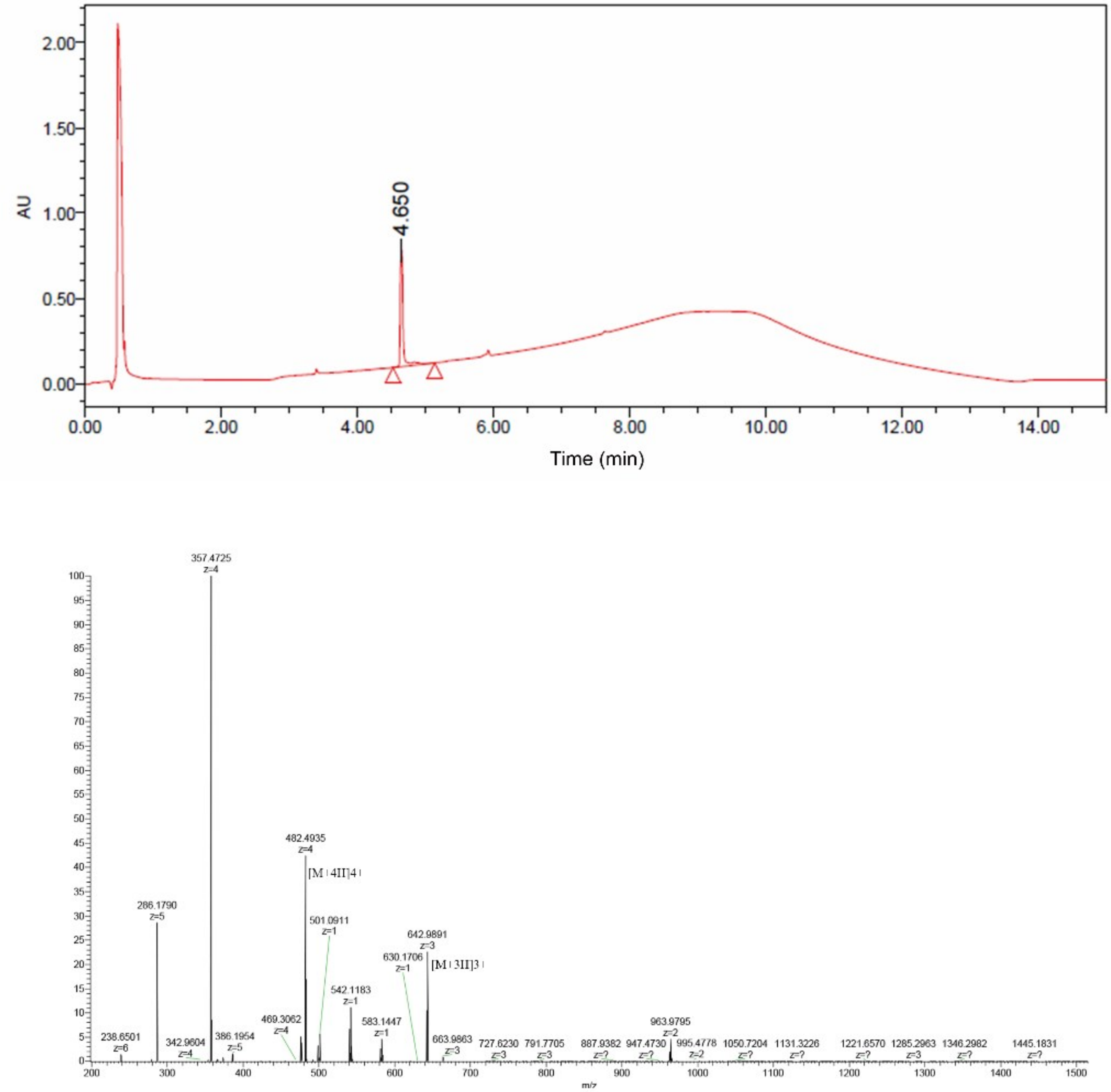

Theoretical MW: 1926.65

Observed MW: 1925.97

Fig S23. UPLC retention time (upper panel) and Mass-spectrum (lower panel) of Ir-cR7.

aR8

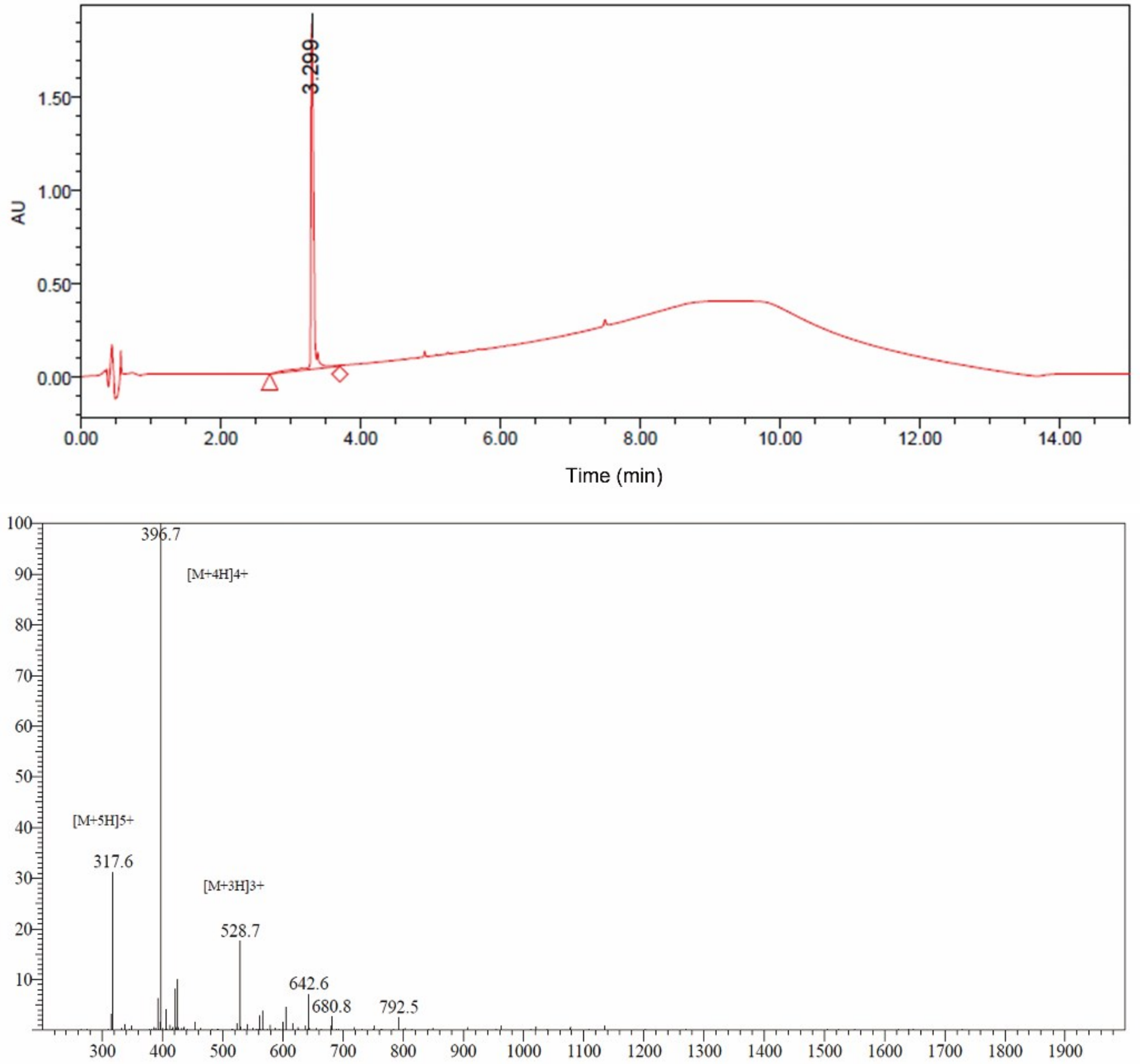

Theoretical MW: 1582.84

Observed MW: 1582.8

Fig S24. UPLC retention time (upper panel) and Mass-spectrum (lower panel) of aR8.

Ir-aR8

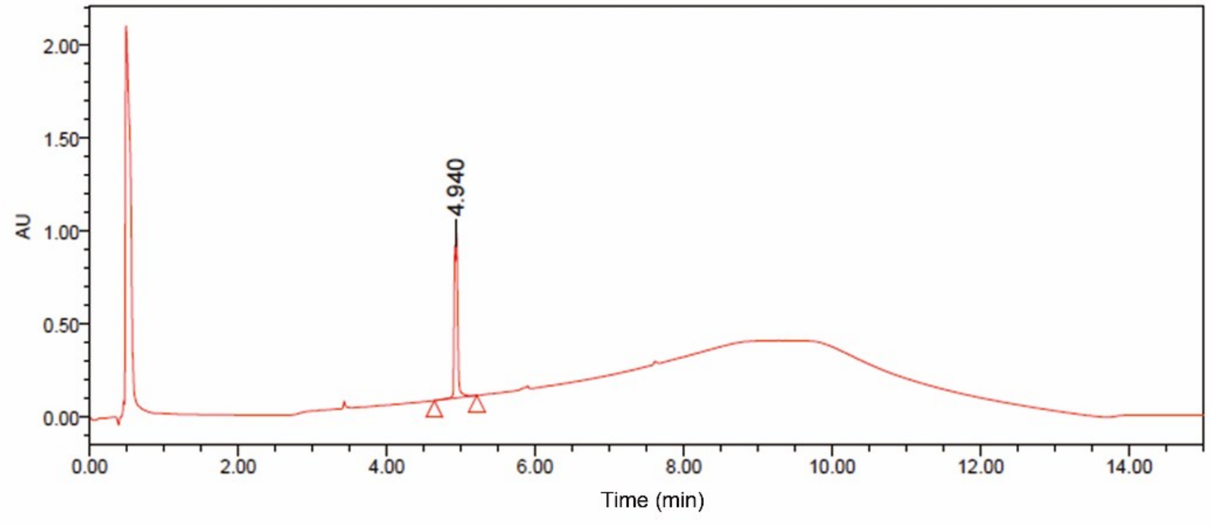

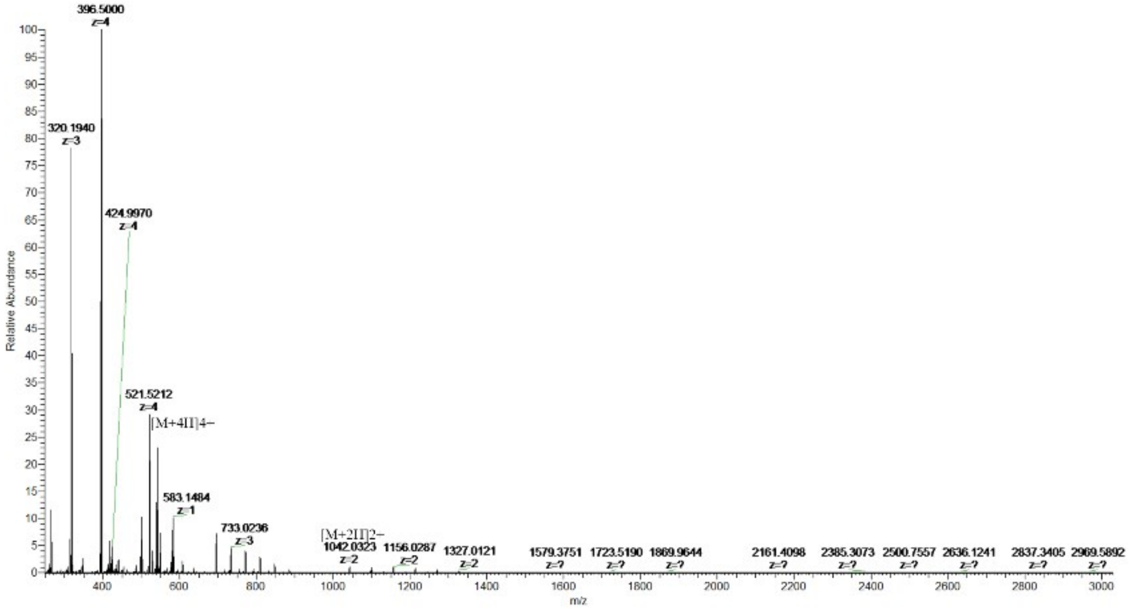

Theoretical MW: 2082.84

Observed MW: 2082.08

Fig S25. UPLC retention time (upper panel) and Mass-spectrum (lower panel) of Ir-aR8.

cR8

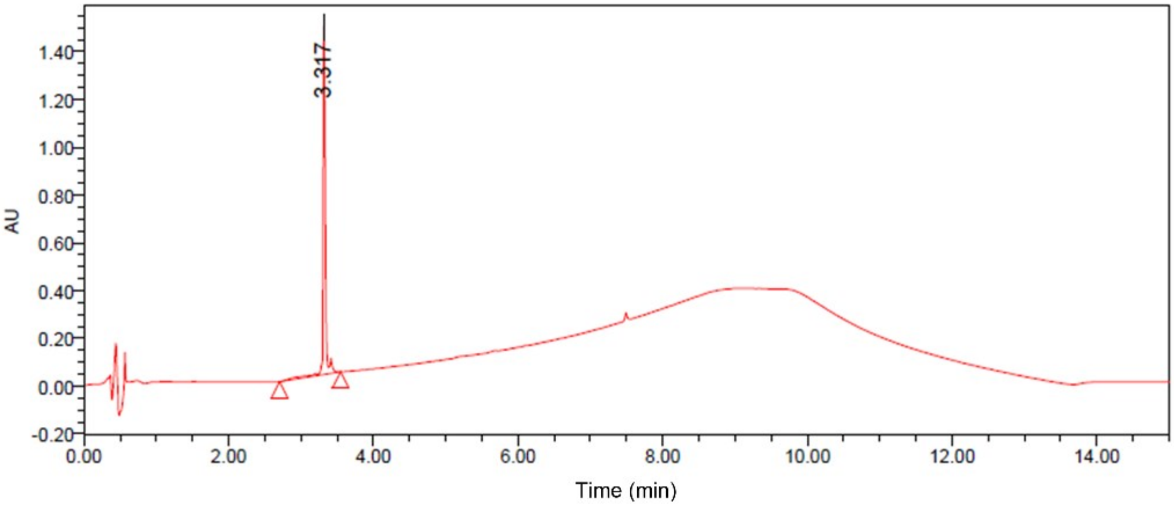

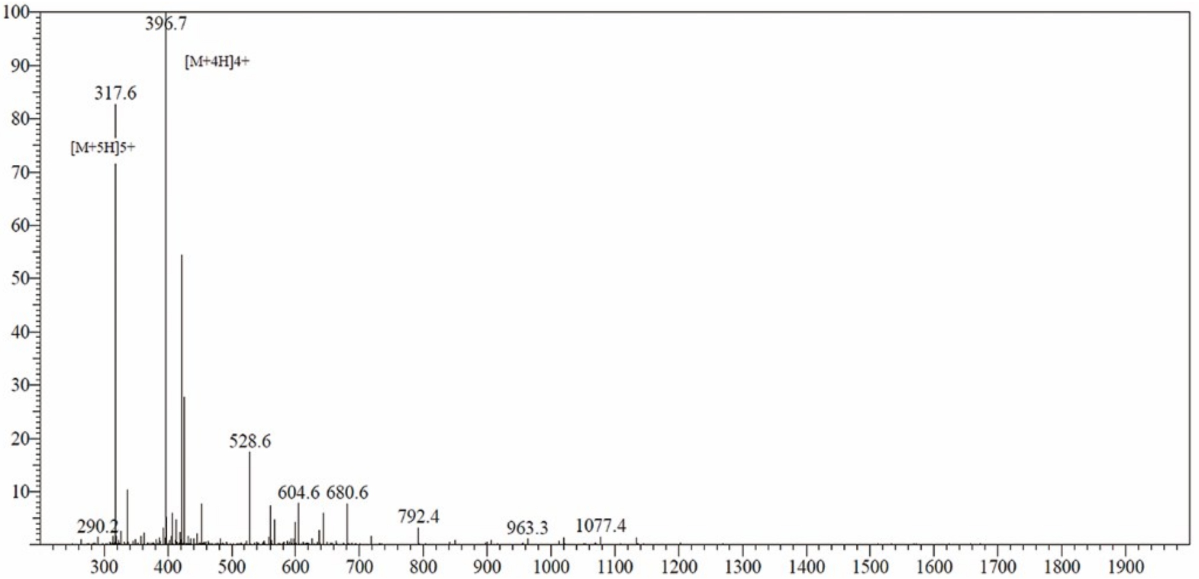

Theoretical MW: 1582.84

Observed MW: 1582.8

Fig S26. UPLC retention time (upper panel) and Mass-spectrum (lower panel) of cR8.

Ir-cR8

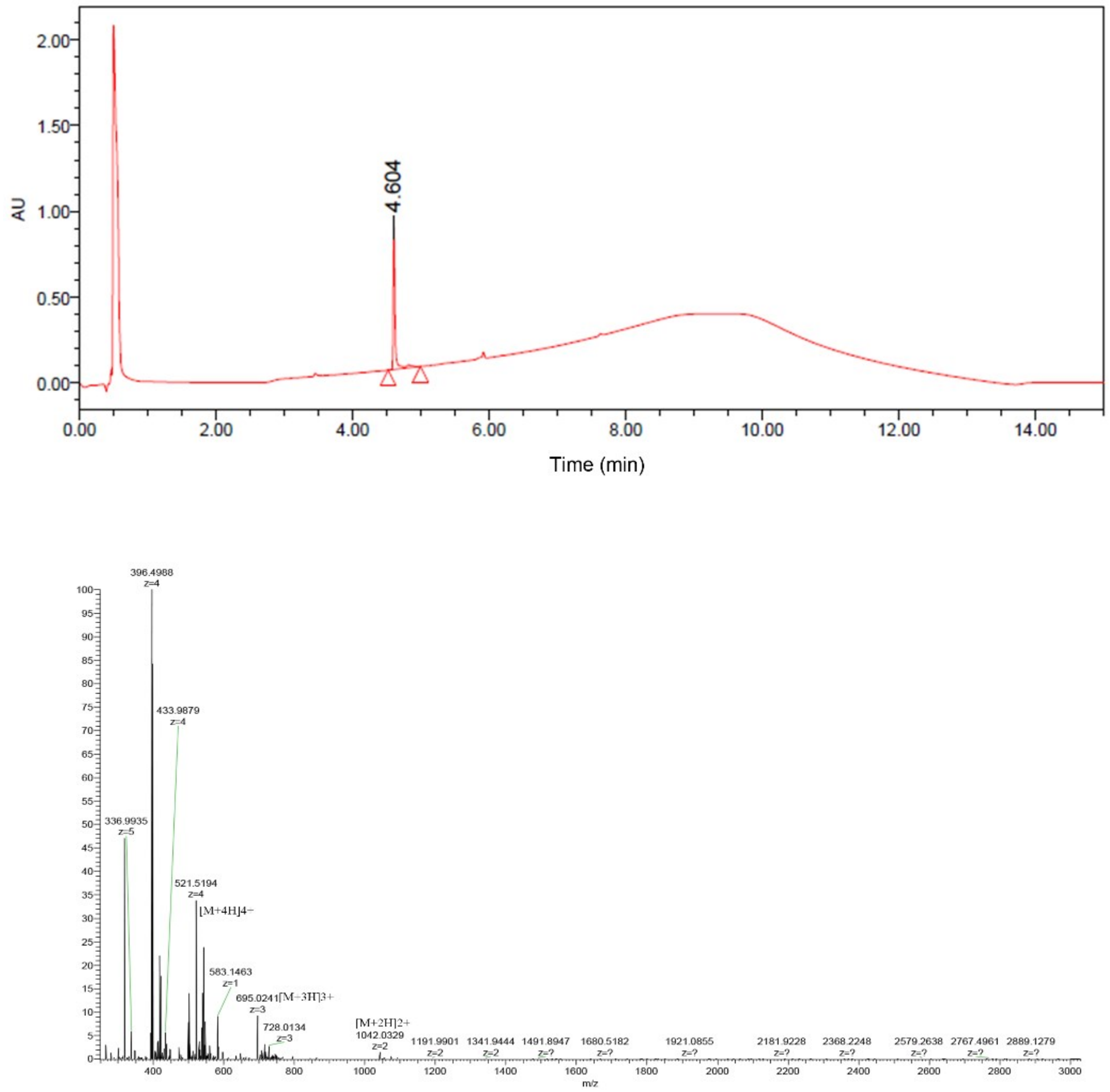

Theoretical MW: 2082.84

Observed MW: 2082.08

Fig S27. UPLC retention time (upper panel) and Mass-spectrum (lower panel) of Ir-cR8.

RL1

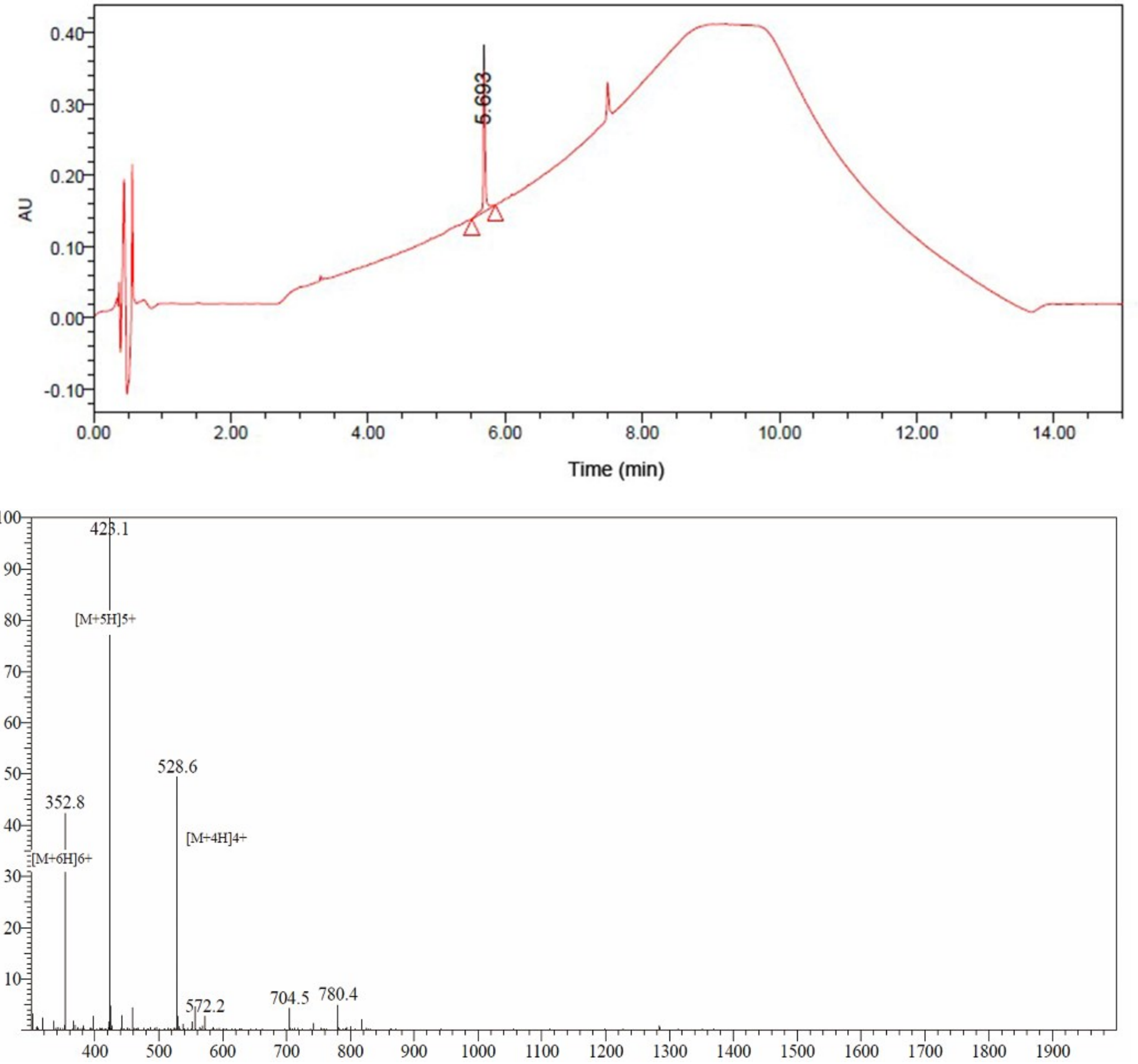

Theoretical MW: 2110.66

Observed MW: 2110.5

Fig S28. UPLC retention time (upper panel) and Mass-spectrum (lower panel) of RL1.

Ir-RL1

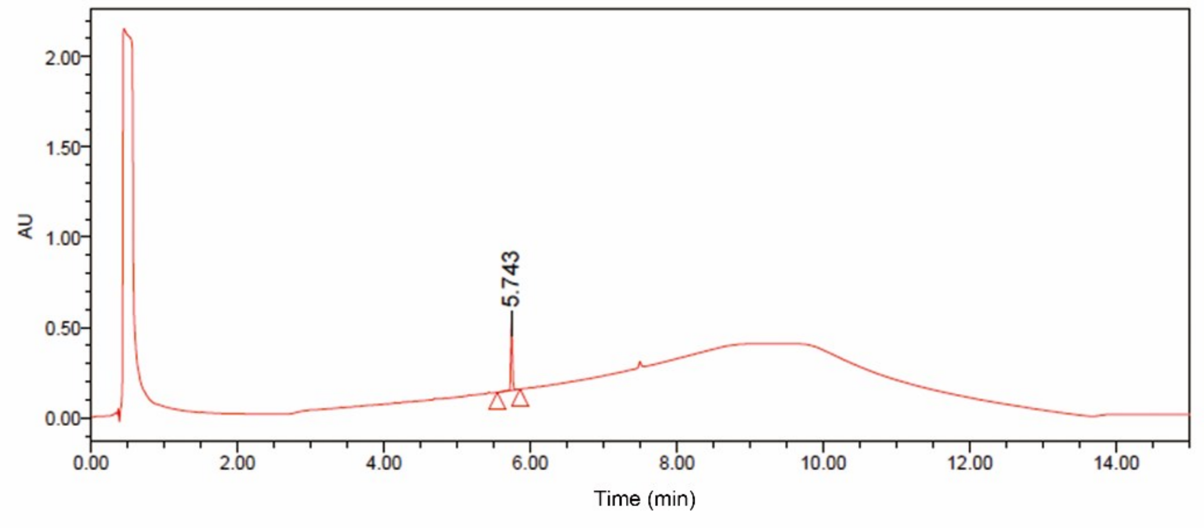

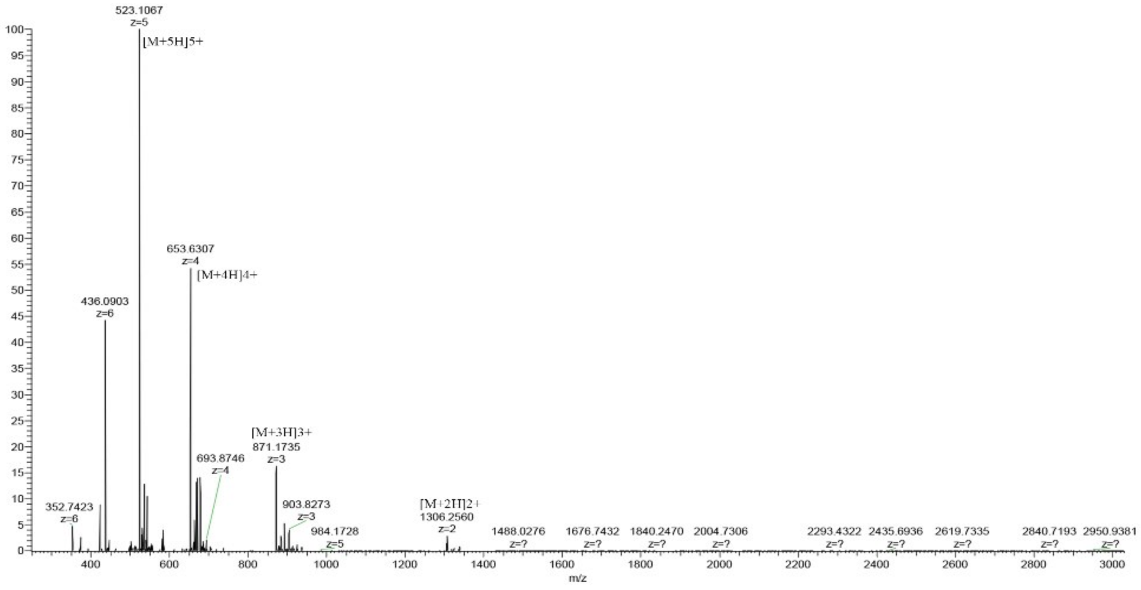

Theoretical MW: 2610.66

Observed MW: 2610.53

Fig S29. UPLC retention time (upper panel) and Mass-spectrum (lower panel) of Ir-RL1.

RL2

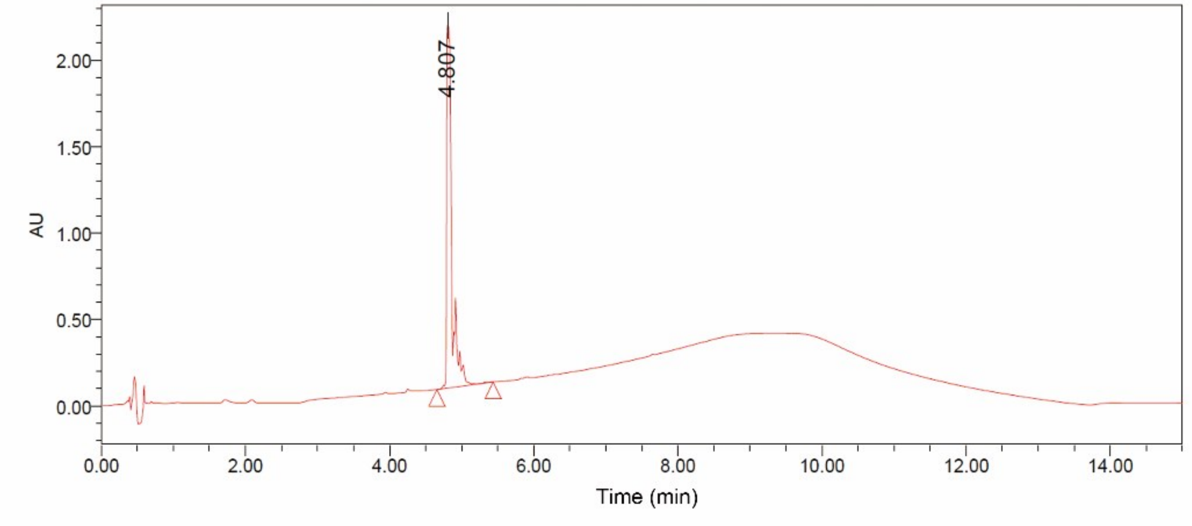

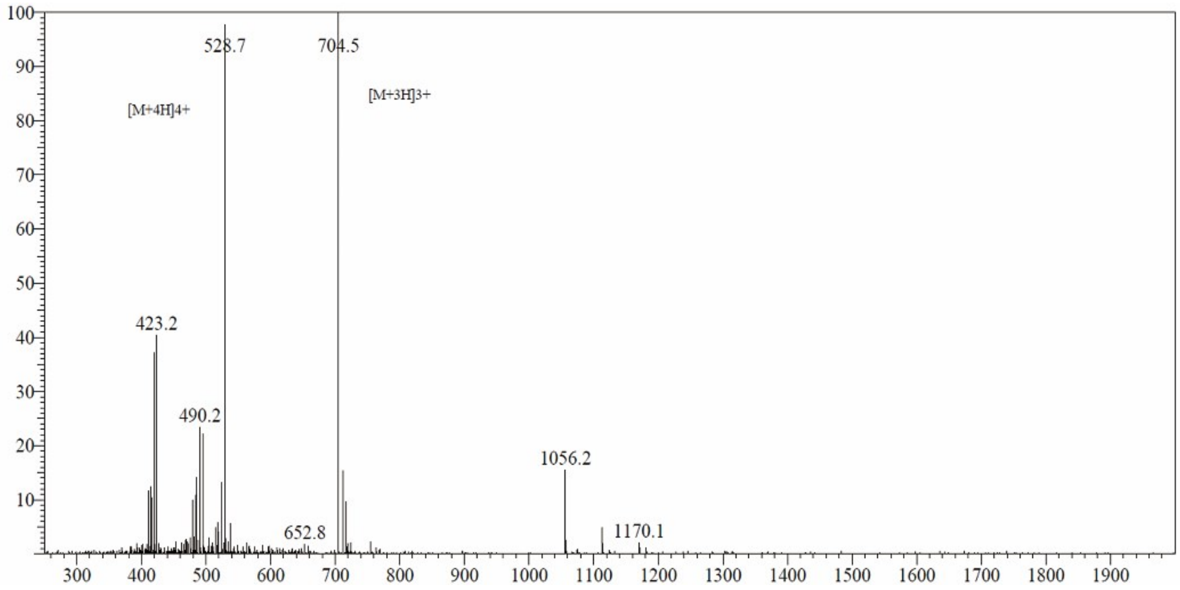

Theoretical MW: 2110.66

Observed MW: 2110.5

Fig S30. UPLC retention time (upper panel) and Mass-spectrum (lower panel) of RL2.

Ir-RL2

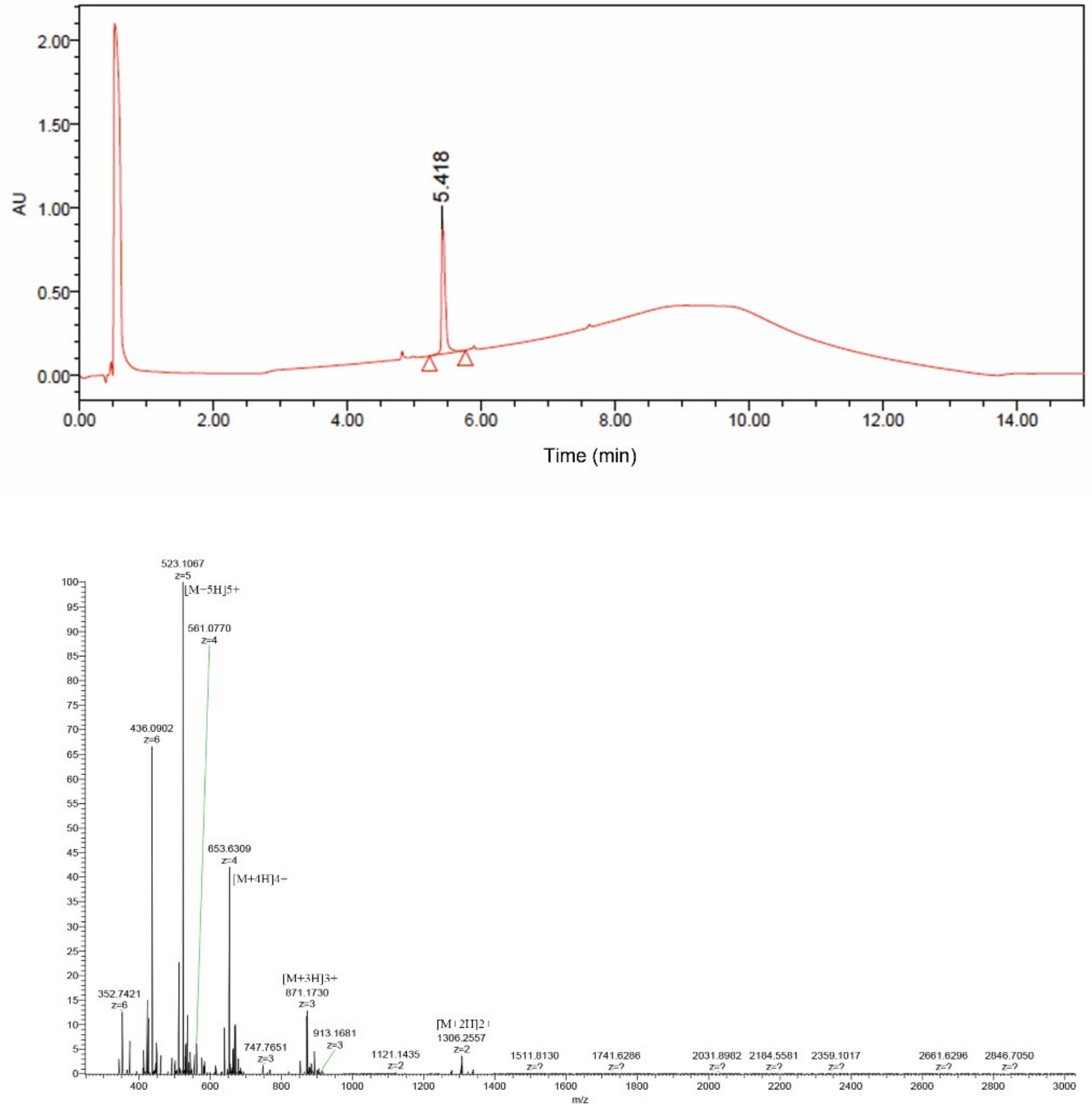

Theoretical MW: 2610.66

Observed MW: 2610.52

Fig S31. UPLC retention time (upper panel) and Mass-spectrum (lower panel) of Ir-RL2.

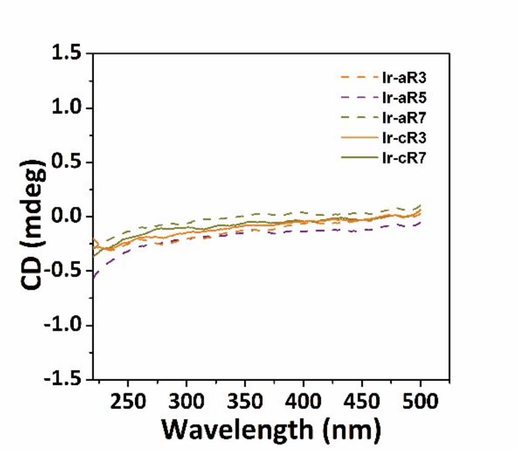

Fig S32. CD spectra of Ir-peptides.

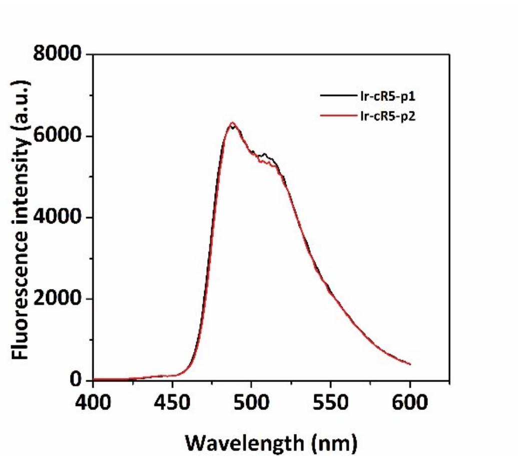

Fig S33. Emission spectra of enantiomers.

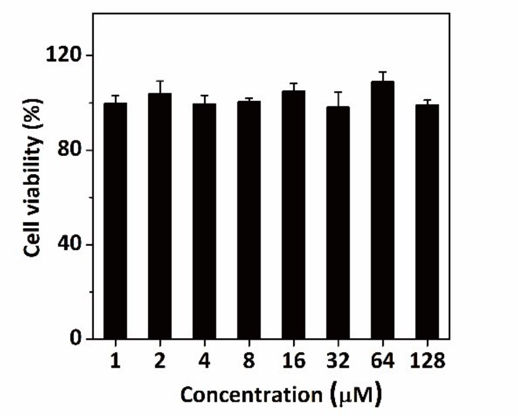

Fig S34. Cytotoxicity assay of $[Ir(ppy)_2(H_2O)_2]OTf$.

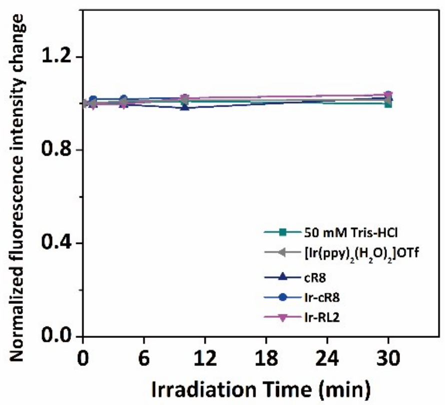

Fig S35. ROS generation of various compounds in 50 mM Tris-HCl.

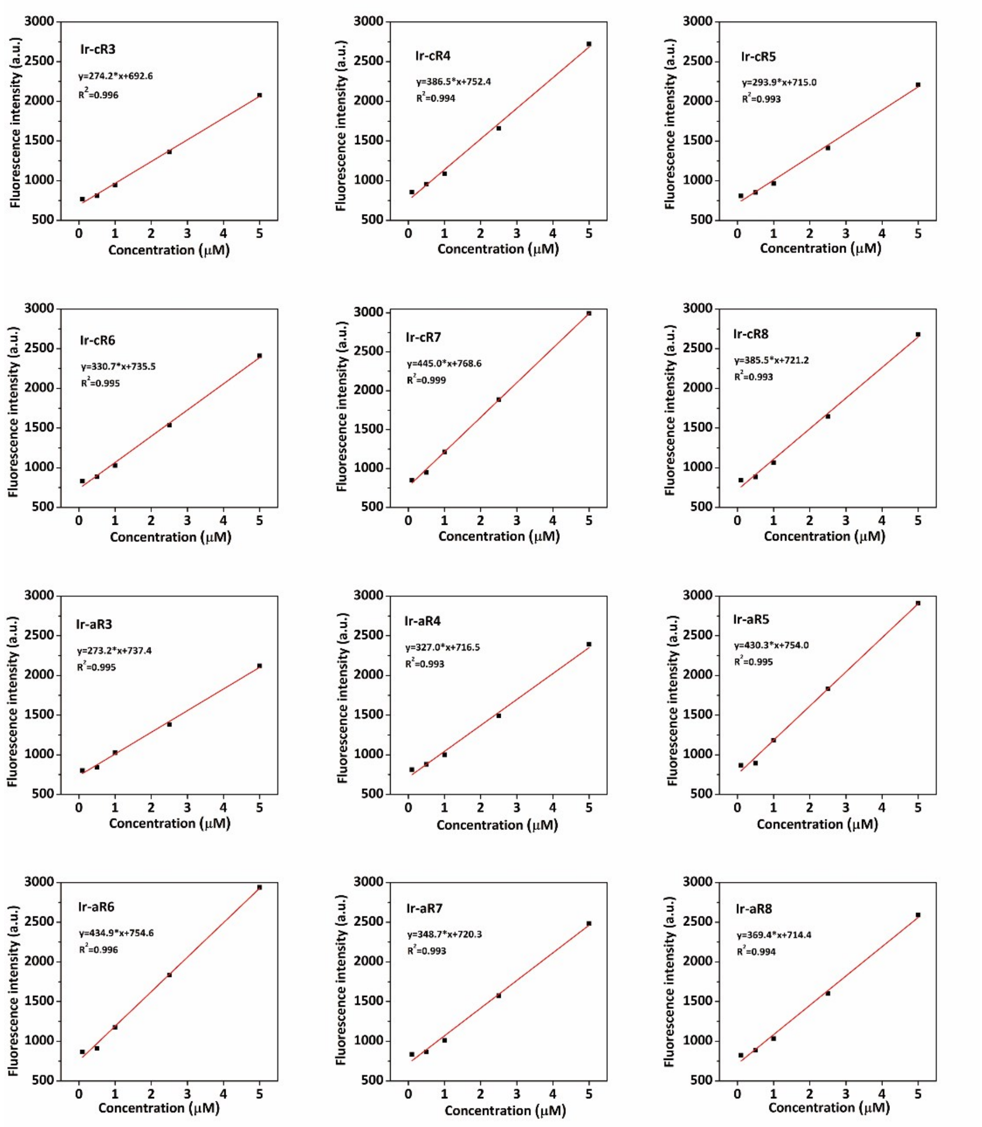

Fig S36. Luminescence-concentration standard curves of Ir-peptides.

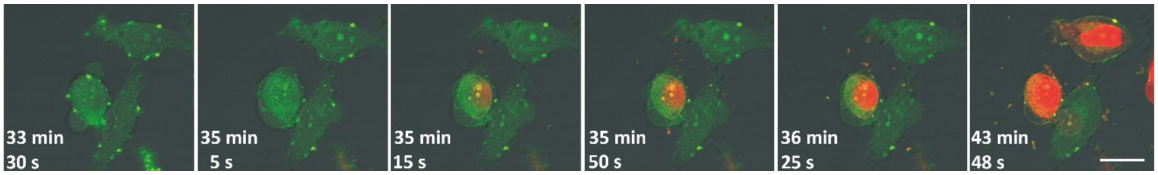

Fig S37. Time-lapse CLSM observation of HeLa cells treated with Ir-aR8(10 μM, green) and PI (red), scale bar, 20 μm.

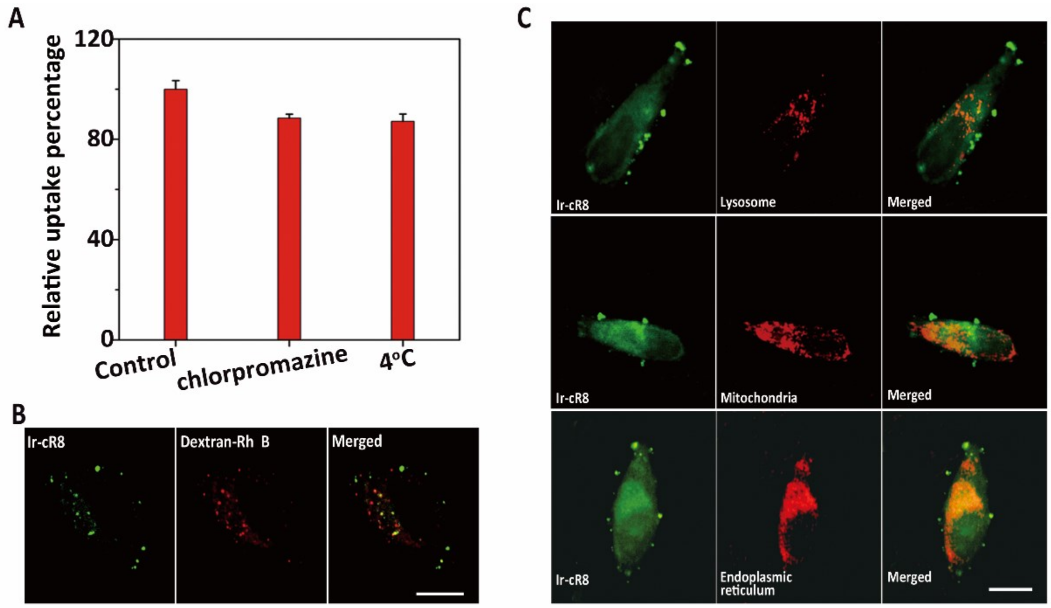

Fig S38. Endocytosis studies suggested an energy-independent pathway when Ir-cR8 enters HeLa cells. (A) Ir-cR8 may not enter cells through energy-dependent clathrin-mediated endocytosis. (B) CLSM image of Hela cells pre-stained with Lyso-Tracker Red DND-99, Mito-Tracker Red, ER-Tracker Red followed by incubation with Ir-cR8 (green). Ir-cR8 had little overlap with lysosomes, mitochondria, and limited overlap with endoplasmic reticulum. Scale bar, 15 μm. (C) CLSM image of Hela cells incubated with Ir-cR8 (green) and Dextran-RhB (red), suggesting Ir-cR8 may not enter cells through macropinocytosis. Scale bar, 20 μm.

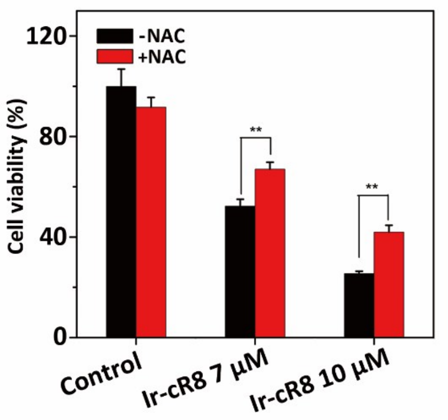

Fig S39. ROS inhibition MTT assay of HeLa cells treated with Ir-cR8 for 24 h, HeLa cells was pre-incubated with NAC (10 mM) for 1 h.

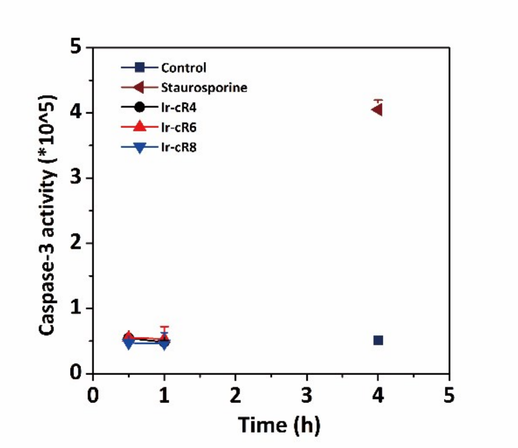

Fig S40. Caspase-3 activity of Ir-cR*n* (*n*=4, 6, 8) with positive (staurosporine)/negative (DMEM) control.

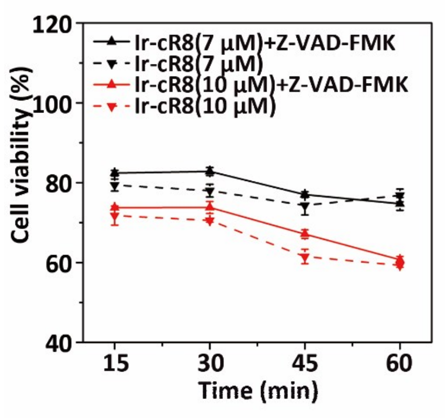

Fig S41. Cytotoxicity induced by Ir-cR8 (7, 10 μM) in an hour with/without caspase inhibitor Z-VAD-FMK (50 μM).

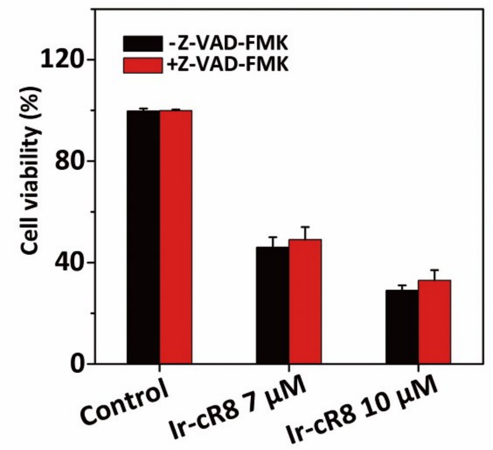

Fig S42. Pan-caspase inhibition MTT assay of HeLa cells treated with Ir-cR8 for 24 h. HeLa cells were pre-incubated with Z-VAD-FMK (50 μM) for 1 h.

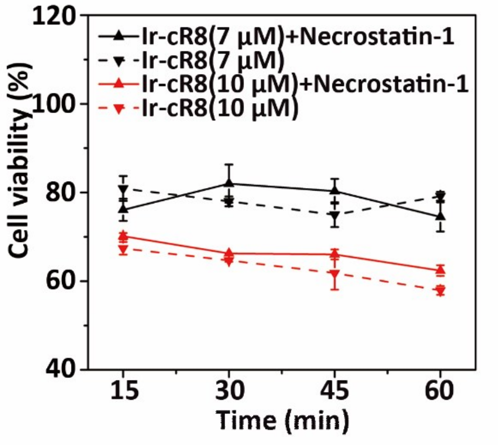

Fig S43. Cytotoxicity induced by Ir-cR8 (7, 10 μM) in an hour with/without necroptosis inhibitor Necrostatin-1 (40 μM).

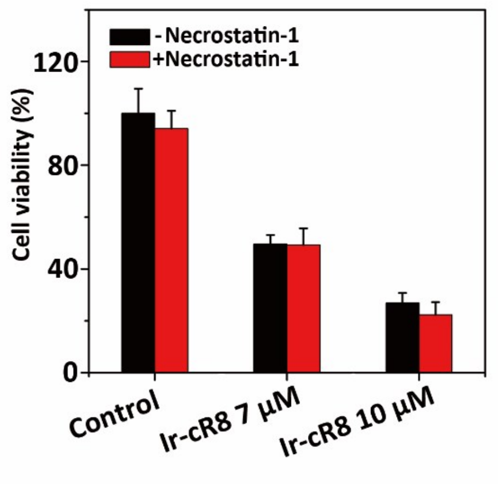

Fig S44. Necroptosis inhibition MTT assay of HeLa cells treated with Ir-cR8 for 24 h. HeLa cells were pre-incubated with necrostatin-1 (40 μM) for 1 h.

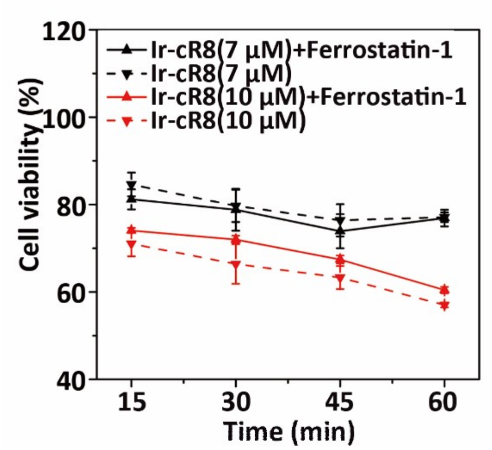

Fig S45. Cytotoxicity induced by Ir-cR8 (7, 10 μM) in an hour with/without ferroptosis inhibitor ferrostatin-1 (5 μM).

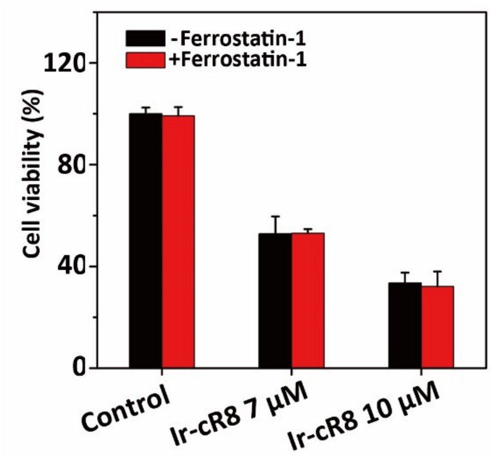

Fig S46. Ferroptosis inhibition MTT assay of HeLa cells treated with Ir-cR8 for 24 h. HeLa cells were pre-incubated with ferrostatin-1 (5 μM) for 1 h.

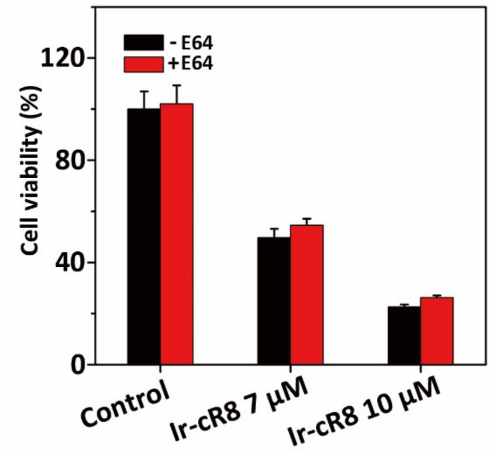

Fig S47. Calpain inhibition MTT assay of HeLa cells treated with Ir-cR8 for 24 h. HeLa cells were pre-incubated with E64 (15 μM) for 1 h.

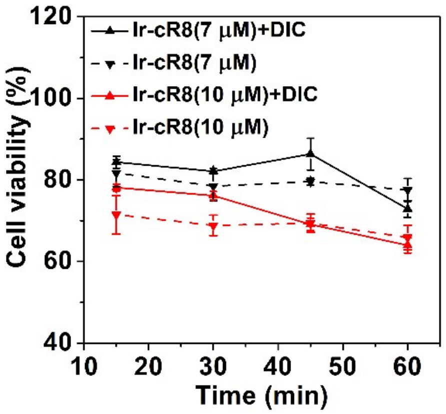

Fig S48. Cytotoxicity induced by Ir-cR8 (7, 10 μM) in an hour with/without pyroptosis inhibitor DIC (10 μM).

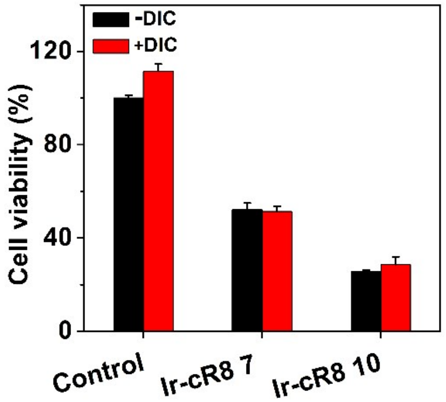

Fig S49. Granzyme A inhibition MTT assay of HeLa cells treated with Ir-cR8 for 24 h. HeLa cells were pre-incubated with DIC (10 μM) for 1 h.

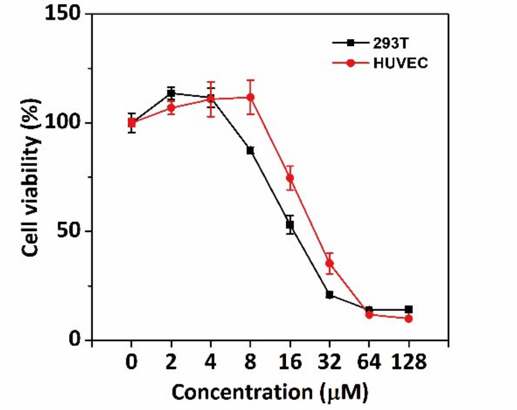

Fig S50. MTT assay of 293T or HUVEC cells treated with Ir-cR8 for 24 h.

Reference:

[1] X. C. Ma, J. L. Jia, R. Cao, X. B. Wang and H. Fei, *J. Am. Chem. Soc.*, 2014, **136**, 17734-17737.
